# The Effectiveness of Power Versus Manual Toothbrushes on Plaque Removal and Gingival Health in Children—A Systematic Review and Meta‐Analysis

**DOI:** 10.1111/idh.12915

**Published:** 2025-07-30

**Authors:** Fatma Dağdeviren, G. A. (Fridus) Van der Weijden, C. P. (Laura) Zijlstra, Dagmar Else Slot

**Affiliations:** ^1^ Department of Periodontology, Academic Centre for Dentistry Amsterdam (ACTA) University of Amsterdam and Vrije Universiteit Amsterdam Amsterdam the Netherlands

**Keywords:** children, electric, gingivitis, manual, plaque, power, toothbrush

## Abstract

**Objective:**

The objective of this systematic review was to assess the efficacy of self‐brushing using a single‐head power toothbrush(PTB) in comparison to a single‐head manual toothbrush(MTB) in terms of plaque removal and reduction of gingivitis in children.

**Materials and Methods:**

MEDLINE‐PubMed and Cochrane CENTRAL were searched up to November 2023. The inclusion criteria comprised randomised clinical trials involving healthy children up to the age of 18 years who did not have fixed orthodontic appliances. Included papers assessed the impact of self‐administered toothbrushing using a rechargeable PTB compared to an MTB on plaque removal and gingivitis. Data extraction was conducted, and the risk of bias was evaluated. A descriptive analysis, a meta‐analysis, and subgroup analysis, when feasible, were carried out.

**Results:**

The search yielded 12 eligible publications, encompassing 30 relevant comparisons. Results showed a significant difference of means (DiffM) on plaque scores in favour of the PTB. Both the end and incremental difference scores indicated a significant difference in effect in favour of the PTB for single‐use brushing (DiffM‐end = −0.26 (95% CI [−0.31; −0.21]; *p* < 0.00001)|DiffM‐difference = −0.26 (95% CI [−0.31; −0.21]; *p* < 0.00001)) and also for follow‐up studies (DiffM‐end = −0.22 (95% CI [−0.36; −0.07]; *p* = 0.004)|DiffM‐difference = −0.34 (95% CI [−0.45; −0.23]; *p* < 0.00001)). The meta‐analysis on gingival index scores showed no significant difference. Subgroup analysis was only possible for the follow‐up studies. For the OR mode of action, a significant difference of means for plaque scores was found (DiffM‐end = −0.19 (95% CI [−0.37; −0.01]; *p* = 0.04)|DiffM‐difference = −0.22 (95% CI [−0.43; −0.01]; *p* = 0.04)). The subgroup analysis contained only studies with a low risk of bias.

**Conclusions:**

There is moderate evidence that in children a PTB offers a small advantage in plaque removal over an MTB. This evidence primarily pertains to PTBs with an OR mode of action.

**Registration:** PROSPERO: #CRD42023144871; ACTA: #2023‐75707

## 1 Introduction

Gingivitis and dental caries are common diseases that affect children [1]. The WHO Global Data on Dental Caries Prevalence in 12‐year‐old children showed that only 17% have a low DMFT [2]. To mitigate the occurrence of dental caries and gingivitis, it is important to manage the accumulation of dental plaque on teeth and gingival tissues [3, 4]. Consequently, routine and effective removal of plaque on a daily basis plays a central part in sustaining optimal oral health [5].

Parents bear the responsibility for their children's oral health care in the early years of life [6]. Establishing good oral hygiene practices is vital, and it is advised that parents initiate toothbrushing as soon as the first tooth emerges in the oral cavity [7, 8]. As children progress in their development, they naturally strive for more independence [6]. Nevertheless, toothbrushing is a fine motor activity and can be challenging for young children [6, 9] due to poor manual dexterity and a lack of motivation [10, 11]. At present, it appears to be advisable for parents to brush their children's teeth at least up to the age of 6 years. Children aged up to 9 years could brush their teeth under parental supervision to ensure effective toothbrushing, and after the age of 9, children may brush their own teeth [8]. Proper cleaning is necessary for the care of erupting and permanent teeth [12], which is challenging for children between 6 and 14 years old, as they experience the mixed dentition stage during the transition from primary to permanent dentition. Under these circumstances, good oral hygiene can be achieved primarily by effective toothbrushing [13]. Hence, it is important to use the right toothbrush and toothbrushing methods [14].

Over the last few decades, powered toothbrushes (PTBs) have become increasingly popular [15]. PTBs have been shown to offer benefits over manual toothbrushes (MTBs) in reducing levels of dental plaque and gingivitis [16]. For adults, the most compelling evidence is available for oscillating–rotating (OR) PTBs [17, 18]. PTBs also appear suitable for children, facilitating the removal of dental plaque to achieve a higher standard of oral hygiene [19]. In the 2014 Cochrane systematic review [20], which compared PTBs and MTBs in terms of effectiveness for plaque removal and gingival health, only 23% of the included studies evaluated their effects in children and adolescents. The review noted that a quarter of adults in the UK claimed to be using a PTB and that its use among children may be even higher [20]. Two recently published systematic reviews indicate that PTBs are more effective than MTBs in plaque removal for patients up to the ages of 13 years [21] and 17 years [22]. It is important to note that these reviews did not differentiate between parental brushing, guided brushing, and self‐brushing [21, 22].

At present, a comprehensive summary of the existing scientific evidence that provides a clear recommendation on the most effective toothbrush for self‐brushing in children is lacking. Such a synthesis would assist oral care professionals in providing informed guidance to young patients and their parents or caregivers. Therefore, the objective of this review is to systematically gather and critically evaluate literature on children engaging in self‐brushing, specifically examining the effectiveness of a PTB compared to an MTB in plaque removal. Additionally, the review considers the impact on reducing gingivitis and explores potential side effects.

## 2 Materials and Methods

This systematic review was prepared and described in accordance with the Cochrane Handbook for Systematic Reviews of Interventions [23], the guidelines provided in Preferred Reporting Items for Systematic reviews and Meta‐Analyses (PRISMA‐statement) [24], and PRISMA Abstracts [24]. The protocol for this review was developed a priori and registered with the International Prospective Register of Systematic Reviews (PROSPERO) [25] under registration number CRD42023144871. The institutional review board of the Academic Centre for Dentistry Amsterdam (ACTA) provided approval for this study under number 2023–75707.

### 2.1 Focused Question

What is the effectiveness of self‐performed toothbrushing with a single‐head PTB compared to a single‐head MTB in terms of plaque removal and gingivitis reduction in children?

### 2.2 Search Strategy

A structured and comprehensive search strategy was designed to retrieve all relevant studies that evaluated the efficacy of toothbrushing in children using either a PTB or an MTB on plaque as the primary parameter of interest. As a secondary parameter of interest, scores of gingivitis were also taken into account. The National Library of Medicine, Washington, D.C. (MEDLINE‐PubMed), and the Cochrane Central Register of Controlled Trials (CENTRAL) were searched for papers relevant to the research question, from inception up to November 2023. Furthermore, the following database sources were searched for possible relevant studies that were either unpublished or published in non‐commercial forms: Open Grey (http://opengrey.eu/), the European Federation of Periodontology (http://efp.org), and the International Association for Dental Research (http://www.iadr.org). There were no restrictions regarding publication year. Finally, the reference lists of the included studies were hand‐searched to identify additional studies that might potentially be relevant. For details regarding the search terms used, see Table 1.

**TABLE 1:** Search terms used for MEDLINE‐PubMed and Cochrane‐CENTRAL. The search strategy was customised according to the database being searched.

The following strategy was used in the search: {<intervention> AND <detail> AND <population>} [intervention] {<(MeSH terms) Toothbrushing OR (text words) Toothbrush> AND [detail] <(text words) Power OR electric> AND [population] <(MeSH terms) Child OR (text words) child*>)

*Note:* The asterisk (*) was used as a truncation symbol.

### 2.3 Screening and Selection

The titles and abstracts of the studies obtained from the searches were screened independently by two reviewers (F.D. and D.E.S.) using the Rayyan [26] web application to select studies that potentially met the inclusion criteria. Only papers written in English were accepted. If eligible aspects were present in the titles and abstracts, the full‐text versions of those potentially relevant papers were obtained. After detailed full‐text reading, these papers were independently categorised (F.D. and D.E.S.) as definitely eligible, definitely not eligible, or questionable. Disagreements concerning eligibility were resolved by consensus, and if disagreement persisted, the issue was resolved through arbitration by a third reviewer (G.A.W.). Papers categorised as “questionable” were evaluated by the same third reviewer (G.A.W.), who made the final decision regarding inclusion. The papers that fulfilled all the inclusion criteria were processed for data extraction.

Inclusion criteria were as follows:

- Randomised controlled clinical trial (RCT)
- Mentally and physically healthy children up to 18 years of age
- No orthodontic appliances
- Self‐performed brushing
- Intervention: single‐head rechargeable PTB (excluding double‐ and triple‐head brushes)
- Control: single‐head MTB (excluding double‐ and triple‐head brushes)
- Outcomes of parameters relevant to the focused question:
  ○ Primary outcome: plaque index scores
  ○ Secondary outcome: gingivitis scores (if presented)

### 2.4 Definition of a Powered Toothbrush

In the dental literature, the terms “electric,” “power,” and “powered” are often used interchangeably to refer to the same type of toothbrushes. A PTB can be broadly defined as a device with a handle incorporating an electromotor that transforms electricity into mechanical action, propelling the brush head. For the purposes of this review, only toothbrushes equipped with rechargeable batteries were taken into consideration. Brushes featuring a replaceable battery to supply electric current and those lacking a moving brush head were not included in the review [27, 28].

### 2.5 Methodological Quality Assessment

Two reviewers (F.D. and C.P.Z.) independently scored the individual methodological qualities of the included studies using the checklist presented in Appendix S1. Studies were classified as having a low risk of bias if they included random allocation, defined inclusion and exclusion criteria, examiner blinding, balanced experimental groups, identical treatment between groups (except for the intervention), and reporting of loss to follow‐up. Studies that properly addressed five of these six criteria were considered to potentially have a moderate risk of bias. If two or more of these six criteria were missing, the study was considered to have a high risk of bias, as proposed by Van der Weijden et al. [29] and described in more detail by Keukenmeester et al. [30]. For this current review, participant blinding was not taken into account, given that it would have been apparent to participants whether they were utilising a PTB or an MTB.

### 2.6 Data Extraction

Eligible studies were processed for data extraction, which involved retrieving details related to the study population, intervention, comparison outcomes, and study characteristics. If available, mean efficacy data concerning baseline, end, and incremental difference together with standard deviations (SDs) were extracted by two reviewers acting independently (F.D. and C.P.Z.) using a specially designed standardised data extraction form. Some studies provided a standard error (SE) of the mean; so, when possible, the authors calculated the SD as follows: SD = √ N*SE. For those papers with missing or incomplete data, the original authors were contacted to request additional data. When there was disagreement between the reviewers regarding any part of the extracted data, the details were discussed until a consensus was reached. If a disagreement persisted, the judgement of a third reviewer (D.E.S.) was decisive.

### 2.7 Data Synthesis

#### 2.7.1 Assessment of Clinical and Methodological Heterogeneity

To assess the clinical heterogeneity of the outcomes across the included studies, the following factors were used: trial design, characteristics of participants, study product, evaluation methods, and industry funding. Methodological heterogeneity was evaluated based on variations in study design and study parameters.

#### 2.7.2 Assessment of Statistical Heterogeneity

As the presence of statistical variability is generally signified by a limited overlap of confidence intervals, the statistical heterogeneity was assessed using the chi‐square test and the *I* ^2^ statistic. A chi‐square test resulting in *p* < 0.1 was considered an indication of significant statistical heterogeneity. As an approximate guide to assessing the degree of inconsistency across studies, an *I* ^2^ statistic of 30%–60% was considered to represent moderate heterogeneity, an *I* ^2^ statistic of 50%–90% substantial heterogeneity, and an *I* ^2^ statistic of 75%–100% considerable heterogeneity [23]. Heterogeneity was considered important when an *I* ^2^ statistic was at least “moderate to substantial” (e.g., *I* ^2^ statistic > 50%).

#### 2.7.3 Descriptive Methods

As a summary, a descriptive data presentation was used for all studies. It was decided a priori to categorise the studies into either single‐use brushing or brushing studies with a follow‐up. Plaque index and gingival index scores were taken into account.

#### 2.7.4 Quantitative Methods

When applicable, quantitative analysis methods, utilising the mean scores and standard deviations extracted from the selected papers, were employed to conduct a meta‐analysis on plaque and gingivitis scores. This analysis was carried out separately for studies focusing on single‐use brushing and for those with a follow‐up. The meta‐analyses were carried out using Review Manager (Version 5.4.1 for Windows; Copenhagen: The Cochrane Collaboration, 2020) in accordance with the suggestions from the PRISMA guidelines [24]. In studies consisting of multiple treatment arms, in which data from one particular group were compared to the data of more than one other group, the number of subjects (*n*) in the group that were entered into the analysis was divided by the number of comparisons. Furthermore, a meta‐analysis was only performed if two or more comparisons could be included. The pooled outcome was expressed as a difference of means (DiffM) with its associated 95% confidence interval (CI). The DiffM between the test and control groups was calculated using both the “random” and “fixed” effects models where appropriate. A fixed‐effect model was used if there were up to three comparisons because estimates of between‐study variance are low for analyses with small numbers of included studies [23, 31]. Data from crossover trials with similar parallel group trials were combined, using the generic inverse variance approach [23, 32] within RevMan [23].

#### 2.7.5 Additional Analyses

To explore possible effect size differences, it was a priori decided to perform subgroup analyses, if possible, for age groups, PTB mode of action, and studies with a low risk of bias, both for single‐use brushing and for studies with a follow‐up, using interaction tests available within RevMan [24].

The testing for publication bias was conducted as proposed by Egger et al. [33]. If the meta‐analysis per outcome involved sufficient trials to make a visual inspection of the plot meaningful (a minimum of 10 trials), funnel plots were used as a tool to assess publication bias. Asymmetry in the inverted funnel plot, signalling potential publication bias, may arise from methodological differences, study heterogeneity, and selective outcome reporting. Additionally, varying quality of reporting and data exclusion practices can contribute to the observed asymmetry [23, 24].

### 2.8 Grading the Body of Evidence

The Grading of Recommendations Assessment, Development, and Evaluation (GRADE) system was used, as proposed by the GRADE working group, to appraise the evidence emerging from this review [34, 35]. Two reviewers (D.E.S. and G.A.W.) rated the quality of the evidence and the strength and direction of the recommendations [36] according to the following aspects: risk of bias, consistency of results, directness of evidence, precision, publication bias, and magnitude of the effect. Any disagreement between the reviewers was resolved through additional discussion.

## 3 Results

### 3.1 Search and Selection Results

**FIGURE 1:**
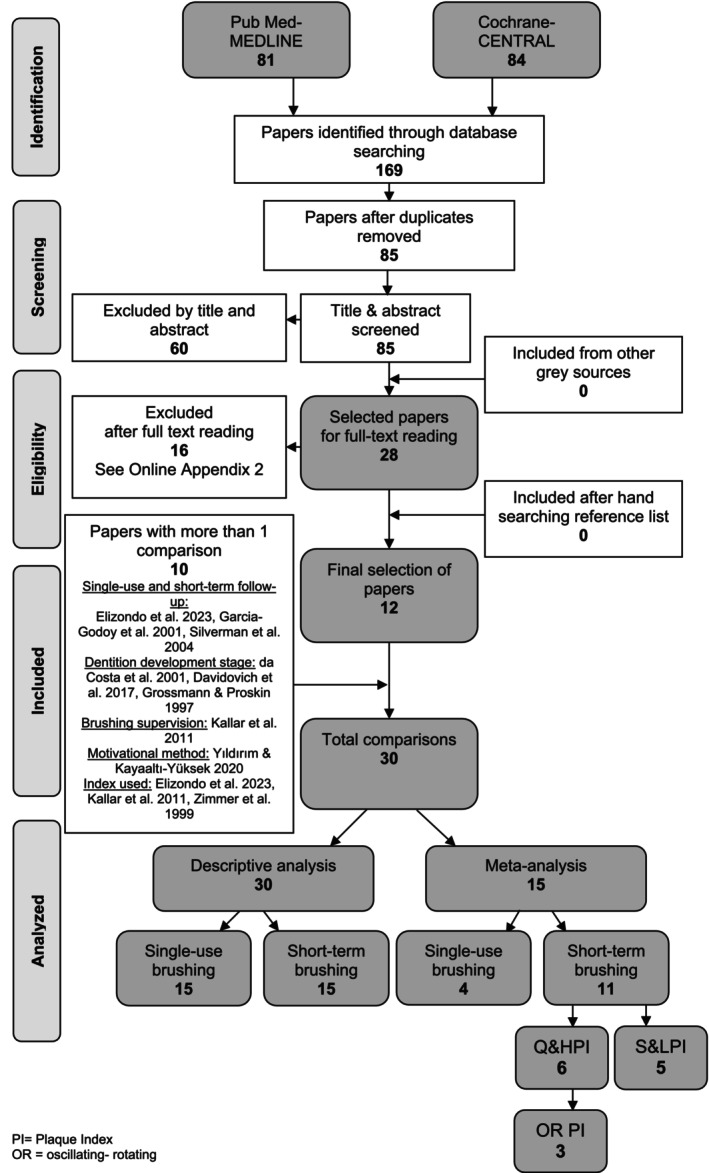
Search and selection results.

A search of the two databases resulted in 85 unique papers (for details, see Figure 1). Screening of the titles and abstracts resulted in 28 papers, which were obtained in full text. Based on a detailed reading of the full text of these papers, 16 papers were excluded (for details, see Appendix S2) and 12 eligible papers were included in this systematic review [37, 38, 39, 40, 41, 42, 43, 44, 45, 46, 47, 48]. No additional eligible papers were included from grey sources or from the reference lists of the selected 12 papers. In total, 30 comparisons were identified. These comprised 15 single‐use brushing comparisons and 15 comparisons with a follow‐up. Three papers [37, 42, 44] described experiments with single‐use brushing as well as with follow‐up. Elizondo et al. [37] evaluated two experiments with a single brushing exercise and one experiment with a follow‐up. Silverman et al. [42] provided one experiment with single‐use brushing and one experiment with a follow‐up. Garcia‐Godoy et al. [44] evaluated three experiments with single‐use brushing and one experiment with a follow‐up.

### 3.2 Assessment of Clinical Heterogeneity

Considerable heterogeneity was observed in the 12 included studies with respect to participants, stages of dentition, and PTB and MTB brands used in the brushing regimen. Information regarding the study characteristics is shown in Table 2.

**TABLE 2:** Overview of the studies processed for data extraction.

Authors (year) (ref) country risk of bias (OAS2)	Study design, washout period (WO) duration blinding oral prophylaxis (PO) plaque accumulation (PA) brushing	No. participants baseline (end) gender mean age age range in years dentition	Groups (brand) mode action	Instructions & regimen	Conclusions of the original authors
I Elizondo et al. (2023) [37] Argentina Moderate	RCT Crossover WO: 4 weeks Single brushing +4 weeks Single‐blind PO:? PA:? after night brushing (baseline) Self‐brushing+NSV	22 (?) ♀:? ♂:? Mean age:? Age range: 4–6 Primary dentition	PTB (Oral‐B Stages Power toothbrush) Oscillation rotation 5600 movements pm MTB (Oral‐B Stages 2 and 3)	Instruction: verbal horizontal brushing technique on paediatric dental typodont Regimen: twice daily Brushing duration: 1 min	The PTB eliminated plaque better throughout 4 weeks of brushing and was more accepted by children compared to the MTB. However, there was no significant difference between PTB and MTB in plaque reduction after single‐use brushing
II Davidovich et al. (2021) [38] Israel Low	RCT Crossover WO: 48 h–1 week Single brushing Single‐blind PO: no PA:? after morning brushing Parental brushing+SV 3–6 years Self‐brushing+SV 7–9 years^ǂ^	42 (41) 3–6 years: 20 (20) 7–9 years: 22 (21) Age range: 3–9 3–6 years: ♀: 14 ♂: 6◊ Mean age: 4.4 7–9 years: ♀: 10 ♂: 12◊ Mean age: 7.8 Primary and mixed dentition^ǂ^	PTB (Oral‐B D100 kids handle with EB10 brush head) Oscillation rotation MTB (Paro Junior Soft #742)	Instruction: verbal and written; MTB in their customary manner Regimen:? Brushing duration:?	The oscillating‐rotating PTB was found to reduce significantly more plaque than the MTB with single‐use brushing in both primary and mixed dentition
III Yıldırım and Kayaaltı‐Yüksek (2020) [39] Turkey Low	RCT Parallel WO:? 15 days Single‐blind PO:? PA:? Self‐brushing SV:?	60 (60) ♀: 29 ♂: 31 Mean age: 8.4 Age rage: 6–12 Primary, mixed and permanent dentition*	Video‐assisted: PTB (Oral‐B Stages Power Mickey Mouse) Oscillation rotation* MTB (Oral‐B Stages)	Instruction: verbal modified Bass technique with video + classical music Regimen: twice daily Brushing duration: 2 min	No significant difference was found between the PTB and the MTB with regard to plaque removal or improvement in gingival health. Also, no significant difference was found between the additional motivational methods used during toothbrushing
			Hourglass‐assisted: PTB (Oral‐B Stages Power Mickey Mouse) Oscillation rotation* MTB (Oral‐B Stages)		
			Control (non‐assisted): PTB (Oral‐B Stages Power Mickey Mouse) Oscillation rotation* MTB (Oral‐B Stages)		
IV Davidovich et al. (2017) [40] Israel Low	RCT Crossover WO: 48 h–1 week Single brushing Single‐blind PO: no PA:? after morning brushing Self‐brushing+SV	41 (41) ♀: 20 ♂: 21 Mean age: 9.0 Age range: 8–11 Mixed and permanent dentition ǂ	PTB (Oral‐B Pro‐Health for me Vitality, D12 kids handle + EB17 soft brush head) Oscillation rotation MTB (Oral‐B Pro‐Expert Cross Action 8+ OK011, soft)	Instruction: verbal and written Regimen:? Brushing duration: PTB 2 min MTB their customary manner	The oscillating‐rotating PTB was found to provide superior plaque reduction relative to an MTB with single‐use brushing in children
V Kallar et al. (2011) [41] India High	RCT Parallel WO:? 12 weeks PO:? PA:? Self‐brushing+SV Self‐brushing+NSV ǂ	200 (?) ♀:? ♂:? Mean age:? Age range: 6–13 Mixed and permanent dentition*	PTB SV (?) MTB SV (?)	Instruction:? Regimen:? Brushing duration:?	The PTB showed significantly more plaque reduction as compared to the MTB group. Supervised groups for both brushes showed a greater plaque reduction
			PTB NSV (?) MTB NSV (?)		
VI Silverman et al. (2004) [42] USA Low	RCT Parallel WO:? Single brushing +6 weeks Single‐blind PO:? PA:24 h Self‐brushing+SV	39 (38) ♀:? ♂:? Mean age:? Age range: 4–5 Primary and mixed dentition*	PTB (Braun Oral‐B Mickey Mouse) Oscillation rotation MTB (Oral‐B Rugrats 20)	Instruction: verbal Regimen: twice daily Brushing duration: 2 min	There were no clinically meaningful differences found between any of the toothbrushes tested during either of the trials with regard to plaque removal or improvement in gingival health
VII da Costa et al. (2001) [43] USA Moderate	RCT Crossover WO: 1 week Single brushing Single‐blind PO: yes PA: 24 h Self‐brushing+SV	29 (?) ♀: 15 ♂: 14 Mean age:? Age range: 4–12 Primary and mixed dentition ǂ	PTB (Braun Oral‐B Ultra plaque remover) Oscillation rotation◊ MTB (Oral‐B Squish Grip)	Instruction:? Regimen:? Brushing duration: 2 min	There were no significant differences concerning plaque removal when the toothbrushes were utilised by children with mixed dentition. PTB promoted significantly greater plaque removal on the lingual surfaces of teeth from children with primary dentition
VIII Garcia‐Godoy et al. (2001) [44] USA Low	RCT Parallel WO:? Single brushing+1 month Single‐blind PO:? PA: 3 × 12–18 h Self‐brushing+SV	70 (66) ♀:? ♂:? Mean age:? Age range: 6–11 Primary and mixed dentition*,^ǂ^	PTB (Braun Oral‐B Kids Power Toothbrush, D10) Oscillation rotation MTB (American Dental Association reference)	Instruction: PTB written and MTB no instruction Regimen: twice daily Brushing duration: 1 min SV At home: 1 min	The PTB is safe for use by children, which has been shown to remove significantly more plaque than an MTB over a period of 30 days' use
IX Zimmer et al. (1999) [45] Germany Low	RCT Crossover WO: 1 week 1 week Single‐blind PO: yes PA:? Self‐brushing SV:?	12 (12) ♀:? ♂:? Mean age:? Age range: 6–12 Primary and mixed dentition*	PTB (Braun plaque control D5525) Oscillation rotation MTB (Elmex super 29)	Instruction: individual instruction per tb Regimen: twice daily Brushing duration: 3 min	No significant difference was found between the PTB and the MTB
X Grossman and Proskin (1997) [46] USA Low	RCT Crossover WO: 1 week Single brushing PO: yes PA: 24 h Self‐brushing+SV	32 (32) ♀:? ♂:? Mean age: 10,6 ◊ Age range: 8–12 Primary and permanent dentition ǂ	PTB (Braun Oral‐B Plaque Remover for Kids) Oscillation rotation* MTB (Reach 6–12, Johnson and Johnson Consumer)	Instruction: Verbal and written Regimen:? Brushing duration: 2 min	The PTB achieved significantly greater plaque removal in both primary and permanent dentition
XI Jongenelis and Wiedemann (1997) [47] The Netherlands Low	RCT Parallel WO:? 1 month PO:? PA:12 h Self‐brushing SV:?	24 (23) ♀: 12 ♂: 12 Mean age: 7.8 ◊ Age range: 5–10 Primary and mixed dentition*	PTB (Dental Logic HP550, Phillips Electronic) Side‐to‐side* MTB (Butler Gum 111, John O. Butler Co.)	Instruction: written Regimen: twice daily Brushing duration: Morning no desired brushing time Evening 2 min	The PTB removes significantly more plaque than the MTB. The differences are the greatest in the posterior lingual areas
XII Strass et al. (1966) [48] USA Moderate	RCT Crossover WO:? PO:? PA:? Self‐brushing SV:?	50 (?) ♀: 19 ♂: 31 Mean age:? Age range: 3.3–6.6◊?	PTB (automatic brush by Dominion Electric Company) Side‐to‐side* MTB (?)	Instruction: verbal Regimen:? Brushing duration:?	In the hands of children 5 years 4 months and above, the PTB was as effective or perhaps slightly more so than the MTB

*Note:* The method of instruction was classified as “none” as reporting normal regimen or no instruction. Instructions according to the manufacturer or written instructions were considered as “written”. Professional instruction by a dental care professional classified as “verbal”. *, defined by the authors of this review; ?, unknown/not provided; ǂ, evaluated separately; ◊, calculated by the authors of this review based on the data presented in the study.

Abbreviations: MTB, manual toothbrush; NSV, no supervision; PA, plaque accumulation; PO, oral prophylaxis; PTB, power toothbrush; SV, supervision.

#### 3.2.1 Study and Subject Characteristics

Four single‐use brushing studies provided information on different dentition development stages. Da Costa et al. [43] and Davidovich et al. [38] evaluated primary and mixed dentitions, Davidovich et al. [40] evaluated mixed and permanent dentitions, and Grossman and Proskin [46] evaluated primary and permanent dentitions.

A variety of plaque indices and their modifications were used. The Quigley & Hein plaque index [49] was used in five papers (II [38], IV [40], VI [42], VIII [44], and XI [47]), Silness and Löe [50] in two papers (III [39] and VII [43]), the global plaque index [51] in one study (X [46]) and IAS technique [52] also in one study (I [37]). Two papers used two plaque indices. One of them (V [41]) recorded plaque both with the Quigley & Hein [49] and the Silness and Löe plaque indices [50] and also differentiated between supervised and non‐supervised brushing. The other paper (IX [45]) used both the Quigley and Hein [49] and the approximal plaque index (API) [53]. One study did not specify the plaque index utilised (XII [48]). Gingivitis was scored in two of the included studies (III [39], VI [42]), which used the Löe & Silness gingival index [54].

The evaluation period varied from single‐use brushing (II [38], IV [40], VI [42], VII [43], VIII [44], and X [46]) to 1 week (IX [45]), 15 days (III [39]), 1 month (I [37], VIII [44], XI [47]), 6 weeks (VI [42]), or 3 months (V [41]), and one study had an unknown duration of follow‐up (XII [48]). Three crossover studies mentioned a washout period of 7 days (VII [43], IX [45], and X [46]), two studies used a period of 48 h to 1 week (II [38], IV [40]), one study used a period of 4 weeks (I [37]), and one study did not report the washout period length (XII [48]). The time frame of plaque accumulation before brushing was 12 h in one study (XI [47]), 12–18 h in another (VIII [44]), and 24 h in three studies (VI [42], VII [43], and X [46]).

#### 3.2.2 Regimen Characteristics

The mode of action of the PTB was an OR movement in nine studies, side‐to‐side in two studies (XI [47] and XII [48]), and unknown in one study (V [41]). The participants in the included studies received a variety of instructions. In nine studies (I [37], II [38], III [39], IV [40], VI [42], VIII [44], X [46], XI [47], and XII [48]) verbal and/or written instructions were provided, where in one study (III [39]) the instructions were accompanied by a video and classical music. In three studies, no information about instruction was given: V [41], VII [43], and IX [45]. In two studies (II [38] and IV [40]) the children brushed without the aid of a mirror. Children in another study (V [41]) were divided into manual or powered brushers. The groups were further divided into two subgroups: supervised brushing and unsupervised brushing. Brushing was completely performed under supervision in six studies: II [38], IV [40], VI [42], VII [43], VIII [44], and X [46]. Study VI [42] specifically stated that the supervisor checked for compliance with the brushing technique. In the supervised studies, the children brushed their teeth away from the blinded examiner.

In studies II [38], V [41], and XII [48], details on toothbrushing duration were not provided. Participants in studies I [37] and VIII [44] were told to brush for 1 min, while in four studies subjects were told to brush for 2 min (III [39], VI [42], VII [43], X [46]), and in one study for 3 min (IX [45]). One study (IV [40]) requested participants to brush with the PTB for 2 min and with the MTB in their customary manner. One study specified no desired duration for morning brushing but prescribed 2 min for evening brushing (XI [47]). In six studies, participants brushed twice daily: I [37], III [39], VI [42], VIII [44], IX [45], and XI [47]. In the other six studies, this information was not provided.

#### 3.2.3 Industry Funding

Funding sources were disclosed in four studies. Two studies (II [38], IV [40]) were funded by Procter & Gamble; another study (VIII [44]) by Oral‐B Laboratories Boston, John O. Butler Company, the American Dental Association, and Procter & Gamble; and a fourth study (XII [48]) by a grant‐in‐aid from the Dominion Electric Company, Mansfield, Ohio.

### 3.3 Assessment of Methodological Heterogeneity

All studies were RCTs. Seven used a crossover design: I [37], II [38], IV [40], VII [43], IX [45], X [46], and XII [48]. Five used a parallel design: III [39], V [41], VI [42], VIII [44], and XI [47].

### 3.4 Methodological Quality Assessment

To estimate the potential risk of bias, the methodological qualities of the included studies are presented in Appendix S1. Based on a summary of the proposed criteria, the potential risk of bias was estimated to be high for Kallar et al. [41], moderate for da Costa et al. [43] and Strass et al. [48], and low for the remaining nine studies.

### 3.5 Study Outcome Results

Appendix S3 (subsections a and b) presents the results of the data extracted per study when the plaque index was used, and Appendix S4 provides the findings when the gingival index was used. The studies are categorised by index and ordered by year of publication. When available, the baseline, end scores, and difference between baseline and end scores with their SDs are presented.

#### 3.5.1 Descriptive Analysis

Table 3a,b presents the descriptive summary of the significant differences in the plaque index and gingival index between the PTB and MTB, as reported by the original authors. Of the 15 comparisons evaluating plaque removal efficacy after a single‐use brushing exercise, seven found significant differences in favour of the PTB. No comparison showed the MTB to be more effective. In total, 15 comparisons evaluated the effectiveness of a PTB compared to an MTB in brushing experiments with a follow‐up. Seven comparisons yielded results in favour of the PTB, seven comparisons produced no significant difference between the PTB and MTB, and for one comparison, no information on plaque index scores was provided. Regarding gingivitis, two studies (III [39] and VI [42]) with altogether four comparisons assessed the gingival index and showed no significant difference between the PTB and MTB.

**TABLE 3:** A descriptive summary of the statistical significance of individual study outcomes related to the effect on plaque removal and gingival health of tooth brushing with a PTB compared to an MTB in children.

Authors (year) (ref) study design, duration per TB	Patient group age group	Intervention		PI	Control	
**(a) Single‐use brushing comparisons**						
Silverman et al. (2004) VI [42] RCT, single brushing	Children 4–5 years	PTB		O	MTB	
Elizondo et al. (2023) I [37] RCT, single brushing	Children 4–6 years	PTB day 0	IAS mm^2^ (50)	O	MTB day 0	
			IAS % (50)	O		
		PTB week 4	IAS mm^2^ (50)	O	MTB week 4	
			IAS % (50)	O		
da Costa et al. (2001) VII [43] RCT, single brushing	Children 4–12 years	PTB P		+	MTB P	
		PTB M		O	MTB M	
Garcia‐Godoy et al. (2001) VIII [44] RCT, single brushing	Children 6–11 years	PTB day 0		+	MTB day 0	
		PTB day 15		O	MTB day 15	
		PTB day 30		O	MTB day 30	
Davidovich et al. (2021) II [38] RCT, single brushing	Children 7–9 years	PTB		+	MTB	
Davidovich et al. (2017) IV [40] RCT, single brushing	Children 8‐11 years	PTB M		+	MTB M	
		PTB P1		+	MTB P1	
Grossman and Proskin (1997) X [46] RCT, single brushing	Children 8–12 years	PTB P		+	MTB P	
		PTB P1		+	MTB P1	
Overall positive for PTB single‐use brushing				7/15 (46.67%)		
**(b) Follow‐up comparisons**						
Strass et al. (1966) XII [48] RCT,?	Children 3.3–6.6◊ years	PTB		?	□	MTB
Silverman et al. (2004) VI [42] RCT, 6 weeks	Children 4–5 years	PTB		O	O	MTB
Elizondo et al. (2023) I [37] RCT, 4 weeks	Children 4–6 years	PTB	IAS mm^2^ (50)	O	□	MTB
			IAS % (50)	+	□	
Jongenelis and Wiedemann (1997) XI [47] RCT, 1 month	Children 5–10 years	PTB		+	□	MTB
Garcia‐Godoy et al. (2001) VIII [44] RCT, 1 month	Children 6–11 years	PTB		+	□	MTB
Yıldırım and Kayaaltı‐Yüksek (2020) III [39] RCT, 15 days	Children 6–12 years	PTB V		O	O	MTB V
		PTB H		O	O	MTB H
		PTB C		O	O	MTB C
Zimmer et al. (1999) IX [45] RCT, 1 week	Children 6–12 years	PTB	S&L (50)	O	□	MTB
			API (53)	O	□	
Kallar et al. (2011) V [41] RCT, 12 weeks	Children 6–13 years	PTB SV	S&L (50)	+	□	MTB SV
			Q&H (49)	+	□	
		PTB NSV	S&L (50)	+	□	MTB NSV
			Q&H (49)	+	□	
Overall positive for PTB short‐term brushing				7/15 (46.67%)	0/4 (0%)	
Overall positive for PTB				14/30 (46.67%)	0/4 (0%)	

*Note:* + = Significant difference in favour of intervention (single‐head toothbrush); − = Significant difference in favour of comparison; o = No significant difference; □ = No data available (Not Tested);? = Unknown/not provided; ◊ = Calculated by the authors of this review based on the presented data in the selected paper.

Abbreviations: API, proximal plaque index; C, control; GI, Gingival Index; H, hourglass‐assisted; M, mixed dentition; MTB, manual toothbrush; NSV, no supervision; P, primary dentition; P1, permanent dentition; PI, Plaque Index; PTB, powered toothbrush; Q&H, Quigley & Hein; S&L, Silness & Löe; SV, supervision; V, video‐assisted.

#### 3.5.2 Side Effects

Five included papers reported on adverse effects and found no evidence of side effects associated with the use of the toothbrush: II [38], IV [40], VIII [44], X [46], and XII [48]. The other included papers did not provide any information on adverse effects (Table 2).

### 3.6 Meta‐Analysis

#### 3.6.1 Overall

A meta‐analysis using mean data from the included studies was possible for the comparison of plaque scores using the Quigley & Hein index and the Silness & Löe index, and for the comparison of gingivitis scores using the Löe & Silness gingival index. Certain studies could not be included in the meta‐analysis because they lacked pre‐ and post‐brushing scores for single‐use brushing (VII [43] and, VIII [44]), lacked baseline and end‐trial data for comparison with a follow‐up (V [41] and XII [48]), or used other plaque indices (I [37], V [41], VII [43], X [46], and XII [48]). Tables 4, 5, 6, 7 provide a summary of the meta‐analysis outcome, and detailed information regarding the meta‐analysis can be found in forest plots presented in Appendices S5–S8. In all studies, the baseline scores were not significantly different (Appendices S5a, S6a, S7a, and S8a). For single‐use brushing studies, a significant effect was found for post‐brushing and incremental difference scores on the plaque index in favour of the PTB (DiffM‐end = −0.26 (95% CI [−0.31; −0.21]; *p* < 0.00001); DiffM‐difference = −0.26 (95% CI [−0.31; −0.21]; *p* < 0.00001)) (Appendix S5b,c). Additionally, in brushing studies with a follow‐up, the Quigley & Hein plaque index end scores were significantly different in favour of the PTB (DiffM‐end = −0.22 (95% CI [−0.36; −0.07]; *p* = 0.004)), as were the incremental difference scores (DiffM‐difference = −0.34 (95% CI [−0.45; −0.23]; *p* < 0.00001)) (Appendix S6b,c). No significant DiffM was found for mean end and incremental difference scores of brushing studies with a follow‐up using the Silness & Löe plaque index and Löe & Silness gingival index (Appendices S7b,c and S8b,c).

**TABLE 4:** Meta‐analysis for single‐use brushing, the pre‐brushing, post‐brushing, and incremental data evaluating PTB compared to MTB on the plaque index score (Q&HPI) reduction in children.

Measurement moment	Included studies # comparisons (ref)	Model	DiffM	Test overall		Test for heterogeneity		
				95% CI	*p*	*I* ^2^ value (%)	*p*	Details in appendix
Pre‐ brushing Q&HPI	3 studies: 4 comparisons; # II [38], IV [40], VI [42]	Random	−0.01	(−0.07; 0.05)	0.74	0	0.53	S5a
Post‐ brushing Q&HPI	3 studies: 4 comparisons; # II [38], IV [40], VI [42]	Random	−0.26	(−0.31; −0.21)	< 0.00001	0	0.75	S5b
Difference Q&HPI	2 studies: 3 comparisons; # II [38], IV [40]	Fixed	−0.26	(−0.31; −0.21)	< 0.00001	0	0.57	S5c

**TABLE 5:** Meta‐analysis for brushing studies with a follow‐up, the baseline, end, and incremental data evaluating a PTB compared to an MTB on the plaque index score (Q&HPI) reduction in children.

Measurement moment	Included studies # comparisons (ref)	Model	DiffM	Test overall		Test for heterogeneity		
				95% CI	*p*	*I* ^2^ value (%)	*p*	Details in appendix
Baseline Q&HPI	4 studies: 4 comparisons; # VI [42], VIII [44], IX [45], XI [47]	Random	0.03	(−0.05; 0.11)	0.51	21	0.28	S6a
End Q&HPI	4 studies: 4 comparisons; # VI [42], VIII [44], IX [45], XI [47]	Random	−0.22	(−0.36; −0.07)	0.004	0	0.81	S6b
Difference Q&HPI	3 studies: 4 comparisons; # V [41], VIII [44], IX [45]	Random	−0.34	(−0.45; −0.23)	< 0.00001	19	0.30	S6c

**TABLE 6:** Meta‐analysis for brushing studies with a follow‐up, the baseline, end, and incremental data evaluating a PTB compared to an MTB on the plaque index score (S&LPI) reduction in children.

Measurement moment	Included studies # comparisons (ref)	Model	DiffM	Test overall		Test for heterogeneity		
				95% CI	*p*	*I* ^2^ value (%)	*p*	Details in appendix
Baseline S&LPI	1 study: 3 comparisons; # III [39]	Fixed	−0.02	(−0.20; 0.16)	0.83	21	0.28	S7a
End S&LPI	1 study: 3 comparisons; # III [39]	Fixed	−0.04	(−0.22; 0.13)	0.64	0	0.80	S7b
Difference S&LPI	2 studies: 5 comparisons; # III [39], V [41]	Random	0.07	(−0.30; 0.45)	0.70	94	< 0.00001	S7c

**TABLE 7:** Meta‐analysis for brushing studies with a follow‐up, the baseline, end, and incremental data evaluating a PTB compared to an MTB on the gingival index score (L&SGI) reduction in children.

Measurement moment	Included studies # comparisons (ref)	Model	DiffM	Test overall		Test for heterogeneity		
				95% CI	*p*	*I* ^2^ value (%)	*p*	Details in appendix
Baseline L&SGI	2 studies: 4 comparisons; # III [39], VI [42]	Random	−0.02	(−0.17; 0.13)	0.78	52	0.10	S8a
End L&SGI	2 studies: 4 comparisons; # III [39], VI [42]	Random	−0.03	(−0.13; 0.08)	0.62	41	0.17	S8b
Difference L&SGI	1 study: 3 comparisons; # III [39]	Fixed	0.02	(−0.07; 0.11)	0.49	56	0.10	S8c

**TABLE 8:** Subgroup analysis for brushing studies with a follow‐up, the baseline, end, and incremental data evaluating an OR mode of action PTB compared to an MTB the plaque index score (Q&HPI) reduction in children.

Measurement moment	Included studies # comparisons (ref)	Model	DiffM	Test overall		Test for heterogeneity		
				95% CI	*p*	*I* ^2^ value (%)	*p*	Details in appendix
Baseline OR Q&HPI	3 studies: 3 comparisons; # VI [42], VIII [44], IX [45]	Fixed	0.00	(−0.01; 0.01)	1.00	0	0.87	S9a
End OR Q&HPI	3 studies: 3 comparisons; # VI [42], VIII [44], IX [45]	Fixed	−0.19	(−0.37; −0.01)	0.04	0	0.72	S9b
Difference OR Q&HPI	2 studies: 2 comparisons; # VIII [44], IX [45]	Fixed	−0.22	(−0.43; −0.01)	0.04	9	0.30	S9c

**TABLE 9:** Subgroup analysis for brushing studies with a follow‐up and low risk of bias, the incremental data evaluating a PTB compared to an MTB, the plaque index score (Q&HPI and S&LPI) reduction in children.

Measurement moment	Included studies # comparisons (ref)	Model	DiffM	Test overall		Test for heterogeneity		
				95% CI	*p*	*I* ^2^ value (%)	*p*	Details in appendix
Difference Q&HPI	2 studies: 2 comparisons; # VIII [44], IX [45]	Fixed	−0.22	(−0.43; −0.01)	0.04	9	0.30	S10a
Difference S&LPI	1 study: 3 comparisons; # III [39]	Fixed	−0.05	(−0.19; 0.08)	0.43	0	0.38	S10b

**TABLE 10:** Estimated evidence profile appraisal of the strength of the recommendation and the direction regarding the use of the PTB compared to the MTB based on plaque removal in self‐brushing children.

Summary of findings table on the body of the estimated evidence profile	
Determinants of quality	Plaque score
Study design (Table 2)	RCT crossover/parallel
#studies *n* = 12 #comparisons	# 12 # 30
Risk of bias (Appendix S2)	Low to high
Consistency (Table 3a,b)	Rather consistent
Directness	Rather generalizable
Precision (Appendices S5–S10)	Rather precise
Reporting bias	Can not be ruled out
Magnitude of the effect (Tables 4, 5, 6, 7, 8, 9)	Small
Strength of the recommendation based on the quality and body of evidence	Moderate
Direction of recommendation	With respect to the plaque removal, there is moderate certainty for a small effect to advise a PTB over an MTB for self‐brushing in children

#### 3.6.2 Additional Analysis

Tables 8 and 9 provide a summary of the subgroup analysis outcome. This was possible for the follow‐up brushing studies with respect to the mode of action. This analysis showed that a PTB using OR technology removed significantly more plaque than an MTB (DiffM‐end = −0.19 (95% CI [−0.37; −0.01]; *p* = 0.04); DiffM‐difference = −0.22 (95% CI [−0.43; −0.01]; *p* = 0.04)) (see Table 8).

A subgroup analysis was conducted on follow‐up brushing studies with a low risk of bias, revealing a significant advantage for the power toothbrush (PTB) in terms of incremental difference scores, as measured by the Quigley & Hein plaque index (DiffM‐difference = −0.22 (95% CI [−0.43; −0.01]; *p* = 0.04)) (see Table 9). Detailed information regarding the forest plots can be found in Appendices S9 and S10.

A test for publication bias could not be performed because the meta‐analysis did not include 10 or more studies, thereby lacking sufficient statistical power for the test to be meaningful [23, 33].

### 3.7 Evidence Profile

Table 10 presents a summary of the various factors that were used to rate the quality of the evidence and the strength of the recommendations, according to the GRADE working group [55]. The risk of bias varied from low to high. The reported outcomes were rather consistent, generalisable, and precise. The magnitude of the effect of using a PTB in comparison to an MTB was rated as a small additional effect. Publication bias could not be ruled out. Taking all factors into consideration, the recommendation was subsequently judged to be moderate.

## 4 Discussion

### 4.1 Summary of Main Findings

This systematic review aimed to compare the clinical effect of a PTB versus an MTB on plaque removal and gingivitis in healthy children. The data emerging from this review were obtained after a comprehensive, systematic literature search. In total, 12 studies were included, which involved 30 comparisons. The efficacy was evaluated descriptively and via a meta‐analysis of the mean clinical outcomes, which included plaque as a primary parameter and gingival inflammation as a secondary parameter. Single‐use brushing studies and brushing studies with a follow‐up were analysed separately. In the meta‐analysis, both designs showed a small but significant benefit in reduction of plaque index scores in favour of the PTB. The descriptive analysis supports this in 46.7% of the cases. However, the magnitude of the difference in effectiveness appears to be small (≈4%–7%). Only two studies assessed gingival health according to the gingival index [52] and showed no significant difference between the PTB and MTB.

### 4.2 Search and Selection Process

Clinical trials that evaluated PTBs with replaceable batteries were excluded from this systematic review. This decision was based on evidence indicating that designs with rechargeable batteries tend to exhibit a more substantial reduction in plaque index scores compared to their replaceable battery‐operated counterparts [27, 28]. Additionally, in alignment with recent findings from a systematic review [56], which reported a lack of clear indications favouring triple‐head over single‐head MTBs for self‐brushing, double‐ and triple‐head toothbrushes were intentionally excluded from consideration in this review.

### 4.3 Design

This review revealed methodological heterogeneity with respect to trial designs that included parallel and crossover designs. The meta‐analysis for single‐use brushing studies combined results from both trial designs. The meta‐analysis for brushing studies with a follow‐up only included parallel design studies on the Silness & Löe plaque index and Löe & Silness gingival index, but combined results from both trial designs on the Quigley & Hein plaque index. The major concern is the risk of bias originating from carry‐over effects in crossover trials [32]. However, given that dental plaque develops in the oral cavity on a daily basis, irrespective of toothbrushing, the aspect of the carry‐over effect in crossover studies is not considered important when mechanical plaque removal is the topic of research. Therefore, combining these designs in the meta‐analysis was deemed appropriate.

### 4.4 Reporting Details

Instructions for toothbrush use were given through various methods such as written instructions, verbal communication, and individual demonstrations, either independently or in combination. Specifics on toothbrushing methods were often not stated. Details on brushing technique, such as how to place the toothbrush on the tooth surface, were missing in the majority of cases. In addition, it was often unclear who provided the instructions. To enhance the reliability and comprehensiveness of interventions, the Template for Intervention Description and Replication (TIDieR) checklist was introduced [57]. This checklist, along with its accompanying guide, aims to enhance the reporting of interventions, streamline authors' descriptions, assist reviewers and editors in their assessments, and facilitate readers in utilising the information. As a recommendation for future research reporting, incorporating the TIDieR checklist in conjunction with CONSORT [58] is encouraged. This review included studies ranging from 1966 to 2023. Among these, four studies (I [37], II [38], III [39], and IV [40]) were published after the introduction of the TIDieR checklist. Although three of them demonstrated a low risk of bias according to the methodological quality assessment, none reported using the TIDieR [57] or CONSORT [58] checklist. Another notable area for improvement in reporting is the identification of funding sources; only four studies (II [38], IV [40], VIII [44], and XII [48]) provided information on funding.

### 4.5 Intervention Details

Reviews [17, 18, 20, 59, 60, 61] have indicated that PTBs with an OR mode of action are more effective at reducing plaque and gingivitis when compared to MTBs. These reviews suggest that OR PTBs outperform those with other modes of action. A possible weakness of the present analysis is that the majority of the included studies involved PTBs with an OR mode of action. Three of the included studies (V [41], XI [47], XII [48]) lacked clarity regarding the mode of action. For two studies (XI [47], XII [48]), this information could be retrieved through an internet search on the brand, revealing that they involved a side‐to‐side motion. However, a subgroup analysis could not be performed on the side‐to‐side mode of action due to a lack of data (XII [48]). Moreover, several brands are currently available with other modes of action, but no eligible publications for these PTBs emerged from the search.

It has been shown that toothbrushing duration is an important factor in the effective removal of dental plaque [6]. It is also known from systematic reviews on toothbrushing in adults that plaque score reduction increases with brushing duration, regardless of the type of toothbrush, whether MTB or PTB [27, 62]. The instructions for brushing time in the present review varied from 1 to 3 min; therefore, not all included studies complied with the 2‐min brushing advice from the American Dental Association (ADA) [63].

### 4.6 Age

Data from the included studies did not allow sub‐analysis based on age or age categories. Effective brushing of teeth by children depends on the brushing technique, motivation, and dexterity (VIII [44]). The dentition in children may consist of primary, erupting, and permanent teeth simultaneously (IV [40]). Generally, children's toothbrushing is deemed inconsistent due to ongoing skill development and challenges in proficiently manoeuvring a toothbrush. The development of manual dexterity is associated with the chronological age of a child. It therefore seems important that children receive professional instruction for using a toothbrush at a young age to achieve optimal results [64]. Parental involvement, in terms of supervision and encouragement to maintain good oral hygiene practices, may also increase compliance (VIII [44]). However, there are inconsistencies in the scientific literature regarding recommendations for the age at which parental involvement in toothbrushing should commence and end. Some suggest initiating toothbrushing from the emergence of the first tooth [8, 9], while others propose starting between ages 1 and 5 [65]. Furthermore, the recommendations on the age at which parents should continue to brush their children's teeth or supervise their brushing exhibit even greater variation. A study evaluating recommendations from 24 dental and paediatric organisations across 10 countries found over 10 different responses regarding the age of parental brushing or supervision [66]. This underscores the existing inconsistencies and the lack of scientific support for this matter. Research investigating variations in plaque removal during self‐brushing across various age groups [9, 67] reveals that younger children typically demonstrate less effective toothbrushing than older children, likely due to less developed brushing skills. For instance, Pujar & Subbareddy [9] showed that plaque index score reduction in 6‐year‐old children was only 57% compared to 82% in 12‐year‐old children. Typically, children achieve effective toothbrushing independence by the age of 11 [68]. However, during adolescence, there is an elevated risk of caries and gingivitis due to changes in oral health and developing health behaviours. Factors such as decreased parental oversight, poor nutrition, increased social needs, and distractions affecting regular brushing motivation contribute to this increased risk [69, 70]. Adolescents commonly exhibit low adherence to oral hygiene, and preventive measures like educational programmes and supervised toothbrushing can enhance oral health [71]. The American Dental Association (ADA) [72], National Health Service (NHS) [73], and *International Association of Paediatric Dentistry (*IAPD) [74] do not give any recommendations on PTB use in children. The American Academy of Paediatrics [75], however, mentions in its paediatric guide to children's oral health that PTBs are especially useful in confined spaces, emphasising their ease of positioning and the small head's ability to control the appropriate amount of toothpaste for children. As the current systematic review showed a slight superiority of PTBs over MTBs in plaque removal for children up to 13 years old, dental care professionals may consider recommending the use of a PTB for self‐brushing in children. Despite differences in inclusion criteria, this aligns with findings from two recently published meta‐analyses comparing PTBs and MTBs in terms of plaque removal in children [21, 22]. This is also consistent with findings from a study excluded from this review, which showed that PTBs demonstrated a greater reduction in plaque and gingivitis than MTBs, even among visually impaired school children [76].

### 4.7 Indices of Gingival Health

While single brushing exercise studies offer valuable insights, it is important to note that their scope may be limited as they may not fully capture the comprehensive benefits for gingival health [28]. To comprehensively assess the effectiveness of PTBs on plaque removal and their potential to enhance gingival health, long‐term measures spanning over 3 months or more are more suitable [11](XII [48]) [77]. The longest follow‐up study that could be included in this systematic review was of 3 months in duration, but it did not evaluate gingival health.

A possible explanation for this lack of information may be that measuring gingival health in children, such as bleeding during stimulation or provocation, is more invasive than assessing plaque index scores [78]. The level of gingival inflammation could be associated with increased pain and discomfort. Individuals with higher levels of gingival inflammation may tend to experience greater discomfort during periodontal probing [79]. This is particularly noteworthy for children, as an unpleasant dental examination or treatment is frequently cited as a primary contributor to dental anxiety [80, 81]. Children tend to harbour a greater apprehension towards invasive procedures compared to non‐invasive approaches [82, 83]. Therefore, a favourable option would be to consider non‐invasive indicators of gingivitis, such as the presence of redness, swelling, and spontaneous bleeding. Only two included papers evaluated gingival health after 15 days (III [39]) and 6 weeks (VI [42]). Although recent work has indicated that there is a clear relationship between 1‐month data and gingival bleeding outcomes at 3 to 6 months [84], longer‐term gingival evaluations of the PTB versus MTB in children appear to be a direction for further research.

### 4.8 Adverse Events

The assessment of toothbrush safety extends to its impact on both hard and soft oral tissues. Out of the 12 studies included in this systematic review, five specifically examined adverse events and uniformly reported an absence of any such occurrences (II [38], IV [40], VIII [44], X [46], XII [48]). A former systematic review [85] evaluated the side effects of OR‐PTBs compared to MTBs. Although the review authors searched for studies in both children and adults, only one [44] of the 35 included papers focused on children. This paper concerning children (VIII [44]) was also included in the present review and the results indicate that the PTB is safe for children, as no gum or tooth abrasion or other adverse events were reported. At present, a large body of published research has consistently shown OR‐PTBs to be safe compared to MTBs, demonstrating that these PTBs do not pose a clinically relevant risk to hard or soft tissues.

However, incidents can still occur. A systematically compiled collection of case reports addressing adverse events linked to the oral use of toothbrushes (both manual and powered) revealed associations with serious adverse events, including ingestion, impaction, immediate trauma, gingival traumatic injury, and seizures arising from toothbrush use. In light of the 118 reported incidents, a significant recommendation underscored that individuals, particularly children, should avoid walking or running with a toothbrush in their mouth [86].

## 5 Conclusions

The results of this review have demonstrated moderately certain evidence for a small but significant difference of means supporting the effectiveness of PTBs compared to MTBs for plaque removal in children. This evidence primarily pertains to PTBs with an OR mode of action. Regarding gingivitis, for which only two studies could be included, the evidence suggests that there is no discernible difference in effectiveness between PTBs and MTBs in children.

## 6 Clinical Relevance

### 6.1 Scientific Rationale for the Study

There is moderate certainty that a PTB is more effective than an MTB with respect to plaque removal and gingivitis reduction in adults. It is important for the dental care professional to know whether the same holds true in children when self‐brushing.

### 6.2 Principal Findings

In children, there is moderate evidence that a PTB provides a small advantage in plaque removal over an MTB. Most data were available for PTBs with an OR mode of action.

### 6.3 Practical Implications

Dental care professionals can recommend the use of a PTB for self‐brushing in children, given its demonstrated efficacy in plaque removal and safety considerations.

## Author Contributions

All authors gave final approval and agreed to be accountable for all aspects of the work ensuring integrity and accuracy. F.D. contributed to the search and selection, analysis and interpretation, and drafted the final version of the manuscript. G.A.V.W. contributed to conception and design, analysis and interpretation, and critically revised the manuscript. C.P.Z. contributed to the design, the preliminary search and selection, analysis and interpretation, and drafted a concept version of the manuscript. D.E.S. contributed to conception and design, search and selection, analysis and interpretation, and critically revised the manuscript.

## Conflicts of Interest

The authors declare no conflicts of interest. Slot and Van der Weijden have formerly received external advisor fees, lecturer fees, or research grants from toothbrush companies. Among these were Braun, Colgate, Dentaid, GABA, Lactona, Oral‐B, Philips, Procter & Gamble, Sara Lee, Tepe, Sunstar, Waterpik, and Unilever.

## Supporting information


Data S1.

## Data Availability

The data that support the findings of this study are available from the corresponding author upon reasonable request.
