# Supplementary material for: The Effectiveness of Power Versus Manual Toothbrushes on Plaque Removal and Gingival Health in Children—A Systematic Review and Meta‐Analysis
Source: Int J Dent Hyg. 2025 Jul 30;23(4):682–702. doi: 10.1111/idh.12915 (PMC12516003; doi:10.1111/idh.12915)
Supplement: Supplementary file 1 — Data S1. [file IDH-23-682-s001.pdf]

To be published in:
International Journal of Dental Hygiene

VERSION:
28 December 2023

Manuscript number:
NA

# The effectiveness of power versus manual toothbrushes on plaque removal and gingival health in children
## *-a systematic review and meta-analysis-*

F. Dağdeviren*
G.A. Van der Weijden*
C.P. Zijlstra*
D.E. Slot*

**Author affiliations:**
*Department of Periodontology, Academic Centre for Dentistry Amsterdam (ACTA), University of Amsterdam and Vrije Universiteit Amsterdam, The Netherlands

**<u>Corresponding author:</u>**
D.E. Slot
Academic Centre for Dentistry Amsterdam (ACTA),
University of Amsterdam and Vrije Universiteit Amsterdam, The Netherlands.
Department of Periodontology
Gustav Mahlerlaan 3004
1081 LA Amsterdam, The Netherlands
Phone: (31)-20-5980-307/174
E-mail: d.slot@acta.nl

**ORCID**

| | |
|---|---|
| F. Dağdeviren | 0000-0002-9092-0438 |
| G.A. Van der Weijden | 0000-0002-5075-8384 |
| C.P. Zijlstra | NA |
| D.E. Slot | 0000-0001-7234-0037 |

**Online Supporting Information Legends**

**Online Appendix S1**

Methodological quality and potential risk of bias scores of the individual included studies.

**Online Appendix S2**

Characteristics of excluded studies after full-text reading.

**Online Appendix S3a**

Mean (SD) plaque index scores categorized per index used PTB compared to MTB in single-use brushing design.

**Online Appendix S3b**

Mean (SD) plaque index scores categorized per index used PTB compared to MTB in brushing studies with a follow-up design.

**Online Appendix S4**

Mean (SD) gingival index scores categorized per index used PTB compared to MTB in brushing studies with a follow-up design.

**Online Appendix S5**

Forest plot using a random and fixed model of the performed meta-analysis for PTB compared to MTB at studies with a single-use brushing on plaque index scores on the Q&HPI in children.

**Online Appendix S6**

Forest plot using a random model of the performed meta-analysis for PTB compared to MTB at studies with a follow-up on plaque index scores on the Q&HPI in children.

**Online Appendix S7**

Forest plot using a fixed and random model of the performed meta-analysis for PTB compared to MTB at studies with a follow-up on plaque index scores on the S&LPI in children.

**Online Appendix S8**

Forest plot using a random and fixed model of the performed meta-analysis for PTB compared to MTB at studies with a follow-up on gingival index scores on the L&SGI in children.

**Online Appendix S9**

Forest plot using a fixed model of the performed subgroup-analysis for the OR mode of action for PTB compared to MTB at studies with a follow-up on plaque index scores on the Q&HPI in children.

**Online Appendix S10**

Forest plot using a fixed model of the performed subgroup analysis for PTB compared to MTB at studies with a follow-up and low risk of bias on plaque index scores on the Q&HPI and S&LPI in children.

**Online Appendix S1**

Methodological quality and potential risk of bias scores of the individual included studies.

| Quality criteria \ Study (year) (ref) | | Elizondo et al. (2023) I (37) | Davidovich et al. (2021) II (38) | Yidirim & Kayaatli-Yüksel (2020) III (39) | Davidovich et al. (2017) I (40) | Kallar et al. (2011) II (41) | Silverman et al. (2004) III (42) | da Costa et al. (2001) IV (43) | Garcia-Godoy et al. (2001) V (44) | Zimmer et al. (1999) VI (45) | Grossman & Proskin (1997) VII (46) | Jongenelis & Wiedemann (1997) VIII (47) | Strass et al. (1966) IX (48) |
|---|---|---|---|---|---|---|---|---|---|---|---|---|---|
| | Study design | Cross-over | Cross-over | Parallel | Cross-over | Parallel | Parallel | Cross-over | Parallel | Cross-over | Cross-over | Parallel | Cross-over |
| Internal validity | Random allocation* | + | + | + | + | + | + | + | + | + | + | + | + |
| | Allocation concealment | ? | + | ? | + | ? | + | ? | ? | + | ? | + | ? |
| | Blinded to product | NA | NA | NA | NA | NA | NA | NA | NA | NA | NA | NA | NA |
| | Blinded to examiner* | + | + | + | + | ? | + | + | + | + | + | + | + |
| | Blinding during statistical analysis | ? | ? | ? | + | ? | ? | ? | ? | ? | ? | ? | ? |
| | Balanced experimental groups* | + | + | + | + | + | + | + | + | + | + | + | + |
| | Reported loss to follow-up* | - | + | + | + | - | + | - | + | + | + | + | + |
| | # (%)of drop-outs | - | 1 (2.4%◊) | 0 | 0 | - | 2 (3,3%◊) | - | 4 (6,1%◊) | 0 | 0 | 1 (4,2%◊) | 0 |
| | Treatment identical except for intervention* | + | + | + | + | + | + | + | + | + | + | + | + |
| External validity | Eligibility criteria defined* | + | + | + | + | - | + | + | + | + | + | + | - |
| | Sample size calculation and power | - | + | + | + | - | + | - | - | - | - | - | - |
| | Point estimates presented for the primary outcome | + | + | + | + | + | + | - | + | + | + | + | + |
| | Measures of variability presented | + | + | + | + | + | + | - | + | - | + | + | - |

| | Quality criteria \ Study (year) (ref) | Elizondo et al. (2023) I (37) | Davidovich et al. (2021) II (38) | Yidirim & Kayaatli-Yüksel (2020) III (39) | Davidovich et al. (2017) I (40) | Kallar et al. (2011) II (41) | Silverman et al. (2004) III (42) | da Costa et al. (2001) IV (43) | Garcia-Godoy et al. (2001) V (44) | Zimmer et al. (1999) VI (45) | Grossman & Proskin (1997) VII (46) | Jongenelis & Wiedemann (1997) VIII (47) | Strass et al. (1966) IX (48) |
|---|---|---|---|---|---|---|---|---|---|---|---|---|---|
| | for the primary outcome | | | | | | | | | | | | |
| | Unit of analysis | Subject | Subject | Subject | Subject | Subject | Subject | Subject | Subject | Subject | Subject | Subject | Subject |
| | Included a per-protocol analysis | ? | + | + | ? | ? | + | - | - | - | - | + | ? |
| | Included an intention-to-treat analysis (ITT) | ? | - | ? | ? | ? | + | - | + | - | - | - | ? |
| Clinical aspects | Validated measurement | + | ? | + | + | + | + | + | + | + | + | + | - |
| | Calibration examiner | + | - | + | + | - | + | + | - | - | - | - | - |
| | Reproducibility data shown | - | - | - | - | - | - | - | - | + | - | - | - |
| Authors' estimated risk of bias | | **M** | **L** | **L** | **L** | **H** | **L** | **M** | **L** | **L** | **L** | **L** | **M** |

Each aspect of the score list was given a rating of '+' for an informative description of the item concerned and a study design meeting the quality standard, '-' for an informative description without a study design that met the quality standard, '?' for lacking or insufficient information and '%' for drop-out of loss to follow-up. When random allocation, defined eligibility criteria, blinding of examiners, balanced experimental groups, identical treatment between groups (except for intervention), and report of follow-up were present, the study was classified as having a low risk of bias. When one of these six criteria was missing, the study was considered to have a moderate potential risk of bias. When two or more of these criteria were missing, the study was considered to have a high potential risk of bias, as proposed by Van der Weijden et al. (2009)(29) ? = not specified/unclear; ◊ = calculated by the authors of this review based on the presented data in the selected; * = reporting criteria for estimating the potential risk of bias; NA= not applicable > not possible for the patient.

**ONLINE Appendix S2.**

Characteristics of excluded studies after full-text reading.

| **Reason for exclusion** | **Study** |
|---|---|
| Battery-powered toothbrush | Borutta 1997, Crawford et al. 1975, Cui et al. 2017, Francis et al. 2021, Ghassemi et al. 2013, Kerr et al. 2019, Mascarenhas et al. 2005, McAllan et al. 1976, Nourallah & Splieth 2004, Owen 1972, Ritsert & Binns 1967, Toto et al. 1966, Yankell & Emling 1996 |
| No self-brushing | Taschner 2012 |
| No comparison | Nowak et al. 2002 |
| Not randomized | Willershausen & Watermann 2001 |

Borutta, A. Plaque removal efficacy of a newly developed powered toothbrush in the primary dentition of pre-school children. *The Journal of Clinical Dentistry,* 1997:8(6), 151-155.

Crawford, A.N., McAllan, L.H., Murray, J.J., & Brook, A.H. Oral hygiene instruction and motivation in children using manual and electric toothbrushes. *Community Dentistry and Oral Epidemiology*, 1975; 3(6), 257-261.

Cui, T.Q., Lin, H.C., Lo, E.C.M., Tao, Y., Zhou, Y., Zhi, Q.H. Randomized clinical trial on the efficacy of electric and manual toothbrushes in plaque removal and gingivitis control in visually impaired school students. *Quintessence international,* 2017; 48(6):481-486.

Francis, M., Hooper, W. J., Worob, D., Huy, G., Santos, S., Goyal, C. R., Qaqish, K., Qaqish, J. G., & Ghassemi, A.. Comparative plaque removal efficacy of a new children's powered toothbrush and a manual toothbrush: Randomized, single use clinical study. *American journal of dentistry*, 2021; 34(6), 338–344.

Ghassemi, A., Vorwerk, L., Hooper, W. Comparative Plaque Removal Efficacy of a New Children's Powered Toothbrush and a Manual Toothbrush. *Journal of Clinical Dentistry,* 2013;24(1):1-4

Kerr, R., Claman, D., Amini, H., Alexy, E., Kumar, A., & Casamassimo, P. S.. Evaluation of the Ability of Five- to 11-Year-Olds to Brush Their Teeth Effectively with Manual and Electric Toothbrushing. *Pediatric dentistry*, 2019; 41(1), 20–24.

Mascarenhas, A.K., Soparkar, P., Al-Mutawa, S., & Udani, T.M. Plaque removal using a battery-powered toothbrush compared to a manual toothbrush. *The Journal of Clinical Dentistry,* 2005; 16(1), 23-25.

McAllan, L.H., Murray, J.J., Brook, A.H., & Crawford, A.N. Oral hygiene instruction in children using manual and electric toothbrushes. Benefits after six months. *British Dental Journal*, 1976; 140(2), 51.

Nourallah, A.W., & Splieth, C.H. Efficacy of occlusal Plaque removal in erupting molars: a comparison of an electric toothbrush and the cross-toothbrushing technique. *Caries research*, 2004; 38(2), 91-94

Owen, T.L. A clinical evaluation of electric and manual toothbrushing by children with primary dentitions. *ASDC Journal of Dentistry for Children,* 1972; 39(1), 15.

Ritsert, E.F., & Binns, W.H. Adolescents brush better with an electric toothbrush. *ASDC Journal of Dentistry for Children,* 1967; 34, 354-358.

Toto, P. D., Goljan, K. R., Evans, J. A., & Sawinski, V. J. A study on the uninstructed use of an electric toothbrush. *Journal of the American Dental Association*, 1966; *72*(4), 904–905.

Yankell, S.L., & Emling, R.C. A thirty-day evaluation of the Rowenta Dentiphant powered toothbrush in children for safety and efficacy. *The Journal of Clinical Dentistry*, 1996; 7(4), 96-100.

Taschner, M., Rumi, K., Mester, A.S., Wei, J., Strate, J., & Pelke, M. Comparing efficacy of plaque removal using professionally applied manual power toothbrushes in 4- to 7-year-old children. *Pediatric dentistry,* 2012; 34(1), 61-65.

Nowak AJ, Skotowski MC, Cugini M, Warren PR. A practice based study of a children's power toothbrush: efficacy and acceptance. *Compendium of Continuing Education in Dentistry*. 2002;23(3 Suppl 2):25-32.

Willershausen, B., & Watermann, L. Longitudinal study to assess the effectively of electric and manual toothbrushes for children. *European Journal of Medical Research*, 2001; 6(1), 39-45.

**ONLINE Appendix S3.**

3a. Mean (SD) plaque index scores categorized per index used PTB compared to MTB in single-use brushing design.

| Study (year) (ref) | Index | GROUP | Baseline | End | Difference |
|---|---|---|---|---|---|
| Davidovich et al. (2021) II (38) | Quigley & Hein plaque index (1962)(49) Turesky et al. (1970) | PTB<br>MTB | 3.32 (0.27♦◊)<br>3.26 (0.27♦◊) | 2.51♦ (0.27♦◊)<br>2.77♦ (0.26♦◊) | -0.77 (0.27◊)<br>-0.51 (0.26◊) |
| Davidovich et al. (2017) IV (40) | Quigley & Hein plaque index (1962)(49) Turesky et al. (1970) | PTB M<br>MTB M | 3.81 (0.24♦◊)<br>3.82 (0.24♦◊) | 3.35♦ (0.17♦◊)<br>3.60♦ (0.17♦◊) | -0.46 (0.17◊)<br>-0.23 (0.17◊) |
| | | PTB P1<br>MTB P1 | 4.15 (0.24♦◊)<br>4.16 (0.23♦◊) | 3.68♦ (0.19♦◊)<br>3.97♦ (0.19♦◊) | -0.48 (0.19◊)<br>-0.19 (0.19◊) |
| Silverman et al. (2004) VI (42) | Quigley & Hein plaque index (1962)(49) Turesky et al. (1970) | PTB<br>MTB | 2.56 (0.35□)<br>2.71 (0.36□) | 1.29 (0.32□)<br>1.44 (0.53□) | -1.27◊ (?)<br>-1.27◊ (?) |
| Garcia-Godoy et al. (2001) VIII (44) | Quigley & Hein plaque index (1962)(49) Turesky et al. (1970) | PTB day 0<br>MTB day 0 | ?<br>? | ?<br>? | -34%<br>-24% |
| | | PTB day 15<br>MTB day 15 | ?<br>? | ?<br>? | -20%<br>-17% |
| | | PTB day 30<br>MTB day 30 | ?<br>? | ?<br>? | -19%<br>-17% |
| da Costa et al. (2001) VII (43) | Silness and Löe (1964)(50) | PTB P<br>MTB P | ?<br>? | ?<br>? | ?<br>? |
| | | PTB M<br>MTB M | ?<br>? | ?<br>? | ?<br>? |
| Grossman & Proskin (1997) X (46) | Global Plaque Index (1973)(50) | PTB P<br>MTB P | 11.99 (4.77)<br>10.02 (3.38) | 4.05 (2.40)<br>5.38 (2.20) | -7.94 (2.98)<br>-4.64 (2.28) |
| | | PTB P1<br>MTB P1 | 15.38 (6.07)<br>14.44 (4.81) | 5.60 (2.73)<br>8.73 (3.24) | -9.78 (3.78)<br>-5.71 (2.87) |

| Study (year) (ref) | Index | GROUP | Baseline | End | Difference |
|---|---|---|---|---|---|
| Elizondo et al. (2023) I (37) | IAS technique (2015)(52) in $mm^2$ | PTB day 0<br>MTB day 0 | 5.84 (3.64♦)<br>4.57 (3.42♦) | 3.49 (4.01♦)<br>2.75 (3.14♦) | -2.41 (2.14♦)<br>-1.83 (2.09♦) |
| | | PTB week 4<br>MTB week 4 | 5.10 (3.36♦)<br>5.51 (2.66♦) | 3.53 (2.43♦)<br>3.54 (2.30♦) | -1.56 (2.47♦)<br>-1.96 (2.33♦) |
| | IAS technique (2015)(52) in % | PTB day 0<br>MTB day 0 | 25.43 (15.73♦)<br>19.12 (13.83♦) | 15.63 (17.31♦)<br>11.70 (12.80♦) | -9.80 (9.52♦)<br>-7.44 (8.57♦) |
| | | PTB week 4<br>MTB week 4 | 22.08 (13.69♦)<br>24.45 (12.08♦) | 16.23 (12.64♦)<br>15.88 (10.35♦) | -5.85 (9.84♦)<br>-8.57 (10.48♦) |

3b. Mean (SD) plaque index scores categorized per index used PTB compared to MTB in <u>brushing studies with a follow-up design</u>.

| Study (year) (ref) | Index | GROUP | Baseline | End | Difference |
|---|---|---|---|---|---|
| Kallar et al. (2011) V (41) | Quigley & Hein plaque index (1962)(49) Turesky et al. (1970) | PTB SV<br>MTB SV | 2.31 (?)<br>2.22 (?) | 1.16 (?)<br>1.48 (?) | -1.14 (0.35□◊)<br>-0.73 (0.21□◊) |
| | | PTB NSV<br>MTB NSV | 2.30 (?)<br>2.29 (?) | 1.38 (?)<br>1.70 (?) | -0.92 (0.49□◊)<br>-0.60 (0.49□◊) |
| Silverman et al. (2004) VI (42) | Quigley & Hein plaque index (1962)(49) Turesky et al. (1970) | PTB<br>MTB | 2.17 (0.42□)<br>2.21 (0.55□) | 1.52 (0.45□)<br>1.75 (0.53□) | -0.65◊ (?)<br>-0.46◊ (?) |
| Garcia-Godoy et al. (2001) VIII (44) | Quigley & Hein plaque index (1962)(49) Turesky et al. (1970) | PTB<br>MTB | 2.64 (0.69)<br>2.57 (0.59) | 2.33 (0.53)<br>2.55 (0.56) | -0.31 (0.58)<br>-0.02 (0.43) |
| Zimmer et al. (1999) IX (45) | Quigley & Hein plaque index (1962)(49) | PTB<br>MTB | 0.00♦ (0.00♦)<br>0.00♦ (0.00♦) | 2.24♦ (0.55♦)<br>2.28♦ (0.44♦) | +2.24♦ (0.55♦)<br>+2.28♦ (0.44♦) |
| Jongenelis & Wiedemann (1997) XI (47) | Quigley & Hein plaque index (1962)(49) Turesky et al. (1970) | PTB<br>MTB | 2.23 (0.26)<br>2.03 (0.26) | 1.20 (0.33)<br>1.48 (0.32) | -1.03◊ (?)<br>-0.55◊ (?) |
| Zimmer et al. (1999) IX (45) | API Lange et al. (1977)(53) | PTB<br>MTB | 0.00♦ (0.00♦)<br>0.00♦ (0.00♦) | 0.93♦ (0.10♦)<br>0.96♦ (0.09♦) | +0.93♦ (0.10♦)<br>+0.96♦ (0.09♦) |
| Kallar et al. (2011) V (41) | Silness and Löe (1964)(50) | PTB SV<br>MTB SV | 1.81 (?)<br>1.79 (?) | 1.91 (?)<br>1.19 (?) | +0.09◊ (0.35□◊)<br>-0.6 (0.42□◊) |
| | | PTB NSV<br>MTB NSV | 1.82 (?)<br>1.82 (?) | 1.09 (?)<br>1.25 (?) | -0.73 (0.42□◊)<br>-0.57◊ (0.57□◊) |
| Yıldırım & Kayaaltı- | Silness and Löe (1964)(50) | PTB V<br>MTB V | 1.29 (0.35)<br>1.14 (0.51) | 0.58 (0.22)<br>0.55 (0.41) | -0.71 (0.28)<br>-0.59 (0.27) |
| | | PTB H | 0.96 (0.41) | 0.52 (0.32) | -0.44 (0.25) |

| Study (year) (ref) | Index | GROUP | Baseline | End | Difference |
|---|---|---|---|---|---|
| Yüksek (2020) III (39) | | MTB H | 0.89 (0.25) | 0.58 (0.28) | -0.31 (0.28) |
| | | PTB C<br>MTB C | 0.88 (0.32)<br>1.07 (0.32) | 0.65 (0.41)<br>0.78 (0.47) | -0.23 (0.25)<br>-0.30 (0.25) |
| Elizondo et al. (2023) I (37) | IAS technique (2015)(52) in mm$^2$ | PTB<br>MTB | 5.84 (3.64♦)<br>4.57 (3.42♦) | 3.53 (2.43♦)<br>3.54 (2.30♦) | -2.31♦ (4.67♦)<br>-1.03♦ (4.47♦) |
| | IAS technique (2015)(52) in % | PTB<br>MTB | 25.43 (15.73♦)<br>19.12 (13.83♦) | 16.23 (12.64♦)<br>15.88 (10.35♦) | -9.20♦ (14.09♦)<br>-3.24♦ (18.03♦) |
| Strass et al. (1966) XII (48) | ? | PTB<br>MTB | ?<br>? | ?<br>? | ?<br>? |

PTB = power toothbrush; MTB = manual toothbrush
SV = supervision; NSV = no supervision
P = primary dentition; M = mixed dentition; P1 = permanent dentition
V = video-assisted; H = hourglass-assisted; C = control
? Unknown/not-provided
□ Not clarified which measure is used
♦ Obtained through data request
◊ Calculated by the authors of this review based on the presented data in the selected paper

**ONLINE Appendix S4.**

Mean (SD) gingival index scores categorized per index used PTB compared to MTB in brushing studies with a follow-up design.

| Study (year) (ref) | Index | GROUP | Baseline | End | Difference |
|---|---|---|---|---|---|
| Silverman et al. (2004) VI (42) | Löe and Silness (1967)(54) | PTB<br>MTB | 0.11 (0.09□)<br>0.14 (0.11□) | 0.05 (0.05□)<br>0.11 (0.11□) | -0.06◊ (?)<br>-0.03◊ (?) |
| Yıldırım & Kayaaltı-Yüksek (2020) III (39) | Löe and Silness (1967)(54) | PTB V<br>MTB V | 0.83 (0.32)<br>0.54 (0.41) | 0.55 (0.25)<br>0.30 (0.35) | -0.28 (0.16)<br>-0.24 (0.18) |
| | | PTB H<br>MTB H | 0.48 (0.35)<br>0.50 (0.22) | 0.23 (0.19)<br>0.29 (0.16) | -0.26 (0.19)<br>-0.21 (0.16) |
| | | PTB C<br>MTB C | 0.37 (0.32)<br>0.61 (0.28) | 0.31 (0.32)<br>0.40 (0.35) | -0.05 (0.16)<br>-0.21 (0.19) |

PTB = power toothbrush; MTB = manual toothbrush; V = video-assisted; H = hourglass-assisted; C = control.

? = Unknown/not-provided

**ONLINE Appendix S5.**

Forest plot using a random and fixed model of the performed meta-analysis for PTB compared to MTB at brushing studies with a <u>single-use brushing</u> on plaque index scores on the Q&HPI (49) in children.

a. Pre-brushing

| | PTB | | | MTB | | | | Mean Difference |
|---|---|---|---|---|---|---|---|---|
| **Study or Subgroup** | **Mean** | **SD** | **Total** | **Mean** | **SD** | **Total** | **Weight** | **IV, Random, 95% CI** |
| Davidovich et al. 2021 | 3.32 | 0.27 | 21 | 3.26 | 0.27 | 21 | 15.2% | 0.06 [-0.10, 0.22] |
| Davidovich et al. M 2017 | 3.81 | 0.24 | 41 | 3.82 | 0.24 | 41 | 37.5% | -0.01 [-0.11, 0.09] |
| Davidovich et al. P1 2017 | 4.15 | 0.24 | 41 | 4.16 | 0.23 | 41 | 39.1% | -0.01 [-0.11, 0.09] |
| Silverman et al. 2004 | 2.56 | 0.35 | 19 | 2.71 | 0.36 | 20 | 8.2% | -0.15 [-0.37, 0.07] |
| **Total (95% CI)** | | | **122** | | | **123** | **100.0%** | **-0.01 [-0.07, 0.05]** |

Heterogeneity: Tau² = 0.00; Chi² = 2.22, df = 3 (P = 0.53); I² = 0%
Test for overall effect: Z = 0.33 (P = 0.74)

Mean Difference
IV, Random, 95% CI

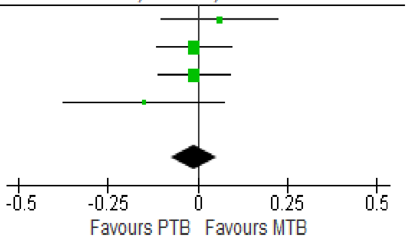


b. Post-brushing

| | PTB | | | MTB | | | | Mean Difference |
|---|---|---|---|---|---|---|---|---|
| **Study or Subgroup** | **Mean** | **SD** | **Total** | **Mean** | **SD** | **Total** | **Weight** | **IV, Random, 95% CI** |
| Davidovich et al. 2021 | 2.51 | 0.27 | 21 | 2.77 | 0.26 | 21 | 10.1% | -0.26 [-0.42, -0.10] |
| Davidovich et al. M 2017 | 3.35 | 0.17 | 41 | 3.6 | 0.17 | 41 | 48.0% | -0.25 [-0.32, -0.18] |
| Davidovich et al. P1 2017 | 3.68 | 0.19 | 41 | 3.97 | 0.19 | 41 | 38.4% | -0.29 [-0.37, -0.21] |
| Silverman et al. 2004 | 1.29 | 0.32 | 19 | 1.44 | 0.53 | 20 | 3.5% | -0.15 [-0.42, 0.12] |
| **Total (95% CI)** | | | **122** | | | **123** | **100.0%** | **-0.26 [-0.31, -0.21]** |

Heterogeneity: Tau² = 0.00; Chi² = 1.19, df = 3 (P = 0.75); I² = 0%
Test for overall effect: Z = 10.11 (P < 0.00001)

Mean Difference
IV, Random, 95% CI

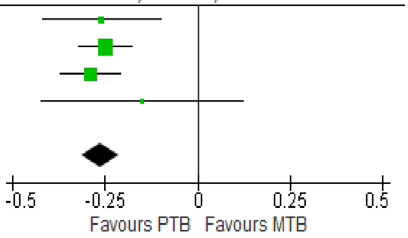


c. Difference

| | PTB | | | MTB | | | | Mean Difference |
|---|---|---|---|---|---|---|---|---|
| **Study or Subgroup** | **Mean** | **SD** | **Total** | **Mean** | **SD** | **Total** | **Weight** | **IV, Fixed, 95% CI** |
| Davidovich et al. 2021 | -0.77 | 0.27 | 21 | -0.51 | 0.26 | 21 | 10.5% | -0.26 [-0.42, -0.10] |
| Davidovich et al. M 2017 | -0.46 | 0.17 | 41 | -0.23 | 0.17 | 41 | 49.7% | -0.23 [-0.30, -0.16] |
| Davidovich et al. P1 2017 | -0.48 | 0.19 | 41 | -0.19 | 0.19 | 41 | 39.8% | -0.29 [-0.37, -0.21] |
| **Total (95% CI)** | | | **103** | | | **103** | **100.0%** | **-0.26 [-0.31, -0.21]** |

Heterogeneity: Chi² = 1.14, df = 2 (P = 0.57); I² = 0%
Test for overall effect: Z = 9.71 (P < 0.00001)

Mean Difference
IV, Fixed, 95% CI

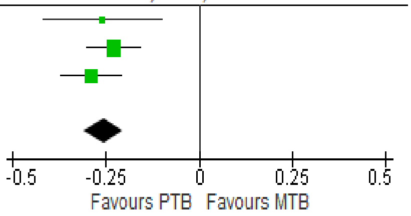

**ONLINE Appendix S6.**

Forest plot using a random model of the performed meta-analysis for PTB compared to MTB at studies with a follow-up on plaque index scores on the Q&HPI (49) in children.

a. Baseline

| | PTB | | | MTB | | | | Mean Difference |
|---|---|---|---|---|---|---|---|---|
| Study or Subgroup | Mean | SD | Total | Mean | SD | Total | Weight | IV, Random, 95% CI |
| Garcia-Godoy et al. 2011 | 2.64 | 0.69 | 35 | 2.57 | 0.59 | 35 | 6.8% | 0.07 [-0.23, 0.37] |
| Jongenelis&Wiedemann 1997 | 2.23 | 0.26 | 12 | 2.03 | 0.26 | 12 | 12.9% | 0.20 [-0.01, 0.41] |
| Silverman et al. 2004 | 2.17 | 0.42 | 19 | 2.21 | 0.55 | 20 | 6.6% | -0.04 [-0.35, 0.27] |
| Zimmer et al. 1999 | 0 | 0.01 | 12 | 0 | 0.01 | 12 | 73.7% | 0.00 [-0.01, 0.01] |
| **Total (95% CI)** | | | **78** | | | **79** | **100.0%** | **0.03 [-0.05, 0.11]** |

Heterogeneity: Tau² = 0.00; Chi² = 3.82, df = 3 (P = 0.28); I² = 21%
Test for overall effect: Z = 0.67 (P = 0.51)

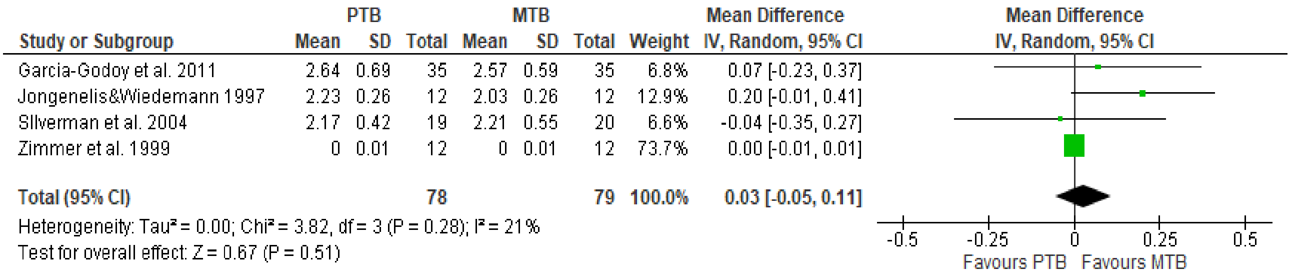


b. End

| | PTB | | | MTB | | | | Mean Difference |
|---|---|---|---|---|---|---|---|---|
| Study or Subgroup | Mean | SD | Total | Mean | SD | Total | Weight | IV, Random, 95% CI |
| Garcia-Godoy et al. 2011 | 2.33 | 0.53 | 34 | 2.55 | 0.56 | 32 | 31.9% | -0.22 [-0.48, 0.04] |
| Jongenelis&Wiedemann 1997 | 1.2 | 0.33 | 12 | 1.48 | 0.32 | 11 | 31.4% | -0.28 [-0.55, -0.01] |
| Silverman et al. 2004 | 1.52 | 0.45 | 18 | 1.75 | 0.53 | 20 | 22.8% | -0.23 [-0.54, 0.08] |
| Zimmer et al. 1999 | 2.24 | 0.55 | 12 | 2.28 | 0.44 | 12 | 13.9% | -0.04 [-0.44, 0.36] |
| **Total (95% CI)** | | | **76** | | | **75** | **100.0%** | **-0.22 [-0.36, -0.07]** |

Heterogeneity: Tau² = 0.00; Chi² = 0.98, df = 3 (P = 0.81); I² = 0%
Test for overall effect: Z = 2.84 (P = 0.004)

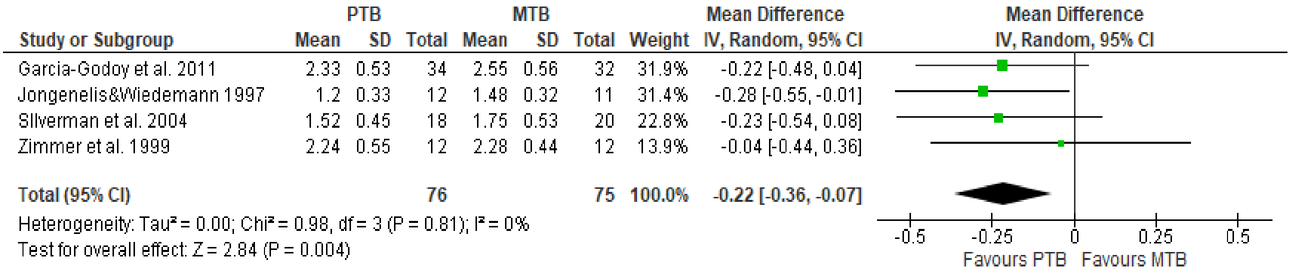


c. Difference

| | PTB | | | MTB | | | | Mean Difference |
|---|---|---|---|---|---|---|---|---|
| Study or Subgroup | Mean | SD | Total | Mean | SD | Total | Weight | IV, Random, 95% CI |
| Garcia-Godoy et al. 2011 | -0.31 | 0.58 | 34 | -0.02 | 0.43 | 32 | 16.6% | -0.29 [-0.54, -0.04] |
| Kallar et al. NSV 2011 | -0.92 | 0.49 | 50 | -0.6 | 0.49 | 50 | 24.9% | -0.32 [-0.51, -0.13] |
| Kallar et al. SV 2011 | -1.14 | 0.35 | 50 | -0.73 | 0.21 | 50 | 51.6% | -0.41 [-0.52, -0.30] |
| Zimmer et al. 1999 | 2.24 | 0.55 | 12 | 2.28 | 0.44 | 12 | 6.9% | -0.04 [-0.44, 0.36] |
| **Total (95% CI)** | | | **146** | | | **144** | **100.0%** | **-0.34 [-0.45, -0.23]** |

Heterogeneity: Tau² = 0.00; Chi² = 3.70, df = 3 (P = 0.30); I² = 19%
Test for overall effect: Z = 6.22 (P < 0.00001)

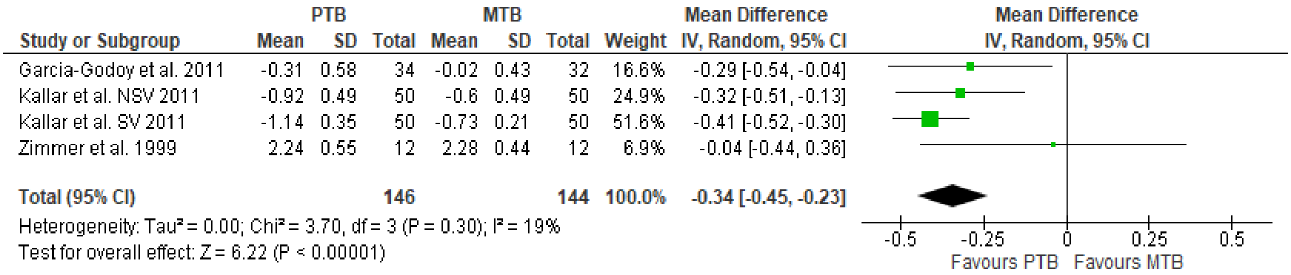

**ONLINE Appendix S7.**

Forest plot using a fixed and random model of the performed meta-analysis for PTB compared to MTB at <u>studies with a follow-up</u> on plaque index scores on the S&LPI (50) in children.

a. Baseline

| | PTB | | | MTB | | | | Mean Difference |
|---|---|---|---|---|---|---|---|---|
| **Study or Subgroup** | **Mean** | **SD** | **Total** | **Mean** | **SD** | **Total** | **Weight** | **IV, Fixed, 95% CI** |
| Yildrim & Kayaalti-Yuksek C 2020 | 0.88 | 0.32 | 10 | 1.07 | 0.32 | 10 | 41.3% | -0.19 [-0.47, 0.09] |
| Yildrim & Kayaalti-Yuksek H 2020 | 0.96 | 0.41 | 10 | 0.89 | 0.25 | 10 | 36.6% | 0.07 [-0.23, 0.37] |
| Yildrim & Kayaalti-Yuksek V 2020 | 1.29 | 0.35 | 10 | 1.14 | 0.51 | 10 | 22.1% | 0.15 [-0.23, 0.53] |
| **Total (95% CI)** | | | **30** | | | **30** | **100.0%** | **-0.02 [-0.20, 0.16]** |

Heterogeneity: Chi² = 2.52, df = 2 (P = 0.28); I² = 21%
Test for overall effect: Z = 0.21 (P = 0.83)

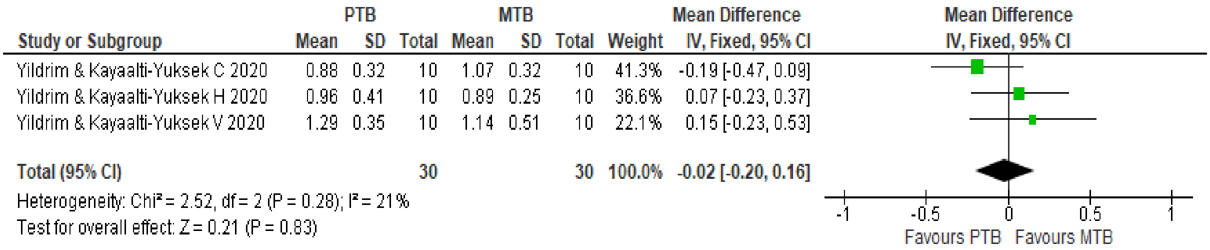


b. End

| | PTB | | | MTB | | | | Mean Difference |
|---|---|---|---|---|---|---|---|---|
| **Study or Subgroup** | **Mean** | **SD** | **Total** | **Mean** | **SD** | **Total** | **Weight** | **IV, Fixed, 95% CI** |
| Yildrim & Kayaalti-Yuksek C 2020 | 0.65 | 0.41 | 10 | 0.78 | 0.47 | 10 | 20.2% | -0.13 [-0.52, 0.26] |
| Yildrim & Kayaalti-Yuksek H 2020 | 0.52 | 0.32 | 10 | 0.58 | 0.28 | 10 | 43.5% | -0.06 [-0.32, 0.20] |
| Yildrim & Kayaalti-Yuksek V 2020 | 0.58 | 0.22 | 10 | 0.55 | 0.41 | 10 | 36.3% | 0.03 [-0.26, 0.32] |
| **Total (95% CI)** | | | **30** | | | **30** | **100.0%** | **-0.04 [-0.22, 0.13]** |

Heterogeneity: Chi² = 0.46, df = 2 (P = 0.80); I² = 0%
Test for overall effect: Z = 0.47 (P = 0.64)

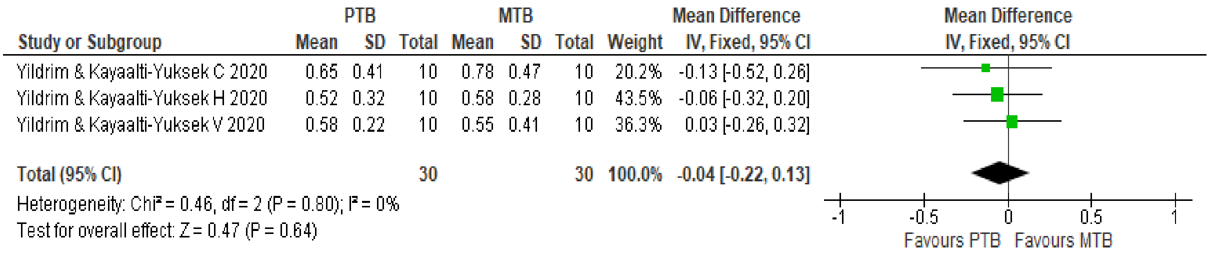


c. Difference

| | PTB | | | MTB | | | | Mean Difference |
|---|---|---|---|---|---|---|---|---|
| **Study or Subgroup** | **Mean** | **SD** | **Total** | **Mean** | **SD** | **Total** | **Weight** | **IV, Random, 95% CI** |
| Kallar et al. NSV 2011 | -0.73 | 0.42 | 50 | -0.57 | 0.57 | 50 | 20.2% | -0.16 [-0.36, 0.04] |
| Kallar et al. SV 2011 | 0.09 | 0.35 | 50 | -0.6 | 0.42 | 50 | 20.6% | 0.69 [0.54, 0.84] |
| Yildrim & Kayaalti-Yuksek C 2020 | -0.23 | 0.25 | 10 | -0.3 | 0.25 | 10 | 19.9% | 0.07 [-0.15, 0.29] |
| Yildrim & Kayaalti-Yuksek H 2020 | -0.44 | 0.25 | 10 | -0.31 | 0.28 | 10 | 19.7% | -0.13 [-0.36, 0.10] |
| Yildrim & Kayaalti-Yuksek V 2020 | -0.71 | 0.28 | 10 | -0.59 | 0.27 | 10 | 19.6% | -0.12 [-0.36, 0.12] |
| **Total (95% CI)** | | | **130** | | | **130** | **100.0%** | **0.07 [-0.30, 0.45]** |

Heterogeneity: Tau² = 0.17; Chi² = 68.78, df = 4 (P < 0.00001); I² = 94%
Test for overall effect: Z = 0.39 (P = 0.70)

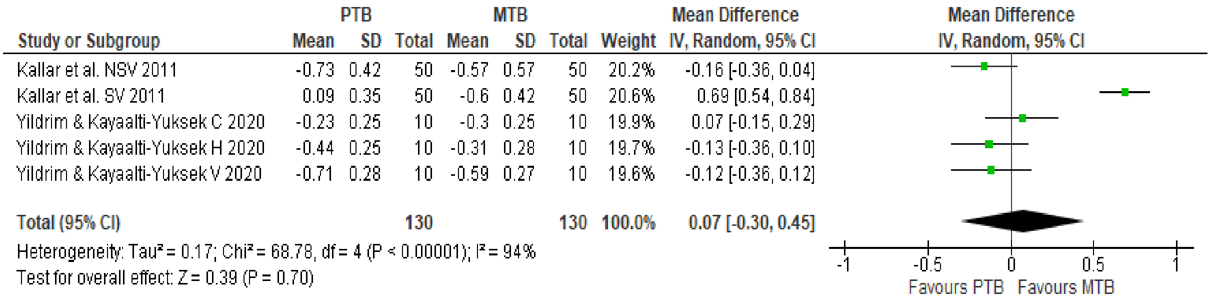

**ONLINE Appendix S8.**

Forest plot using a random and fixed model of the performed meta-analysis for PTB compared to MTB at <u>studies with a follow-up</u> on gingival index scores on the L&SGI (54) in children.

a. Baseline

| Study or Subgroup | PTB Mean | PTB SD | PTB Total | MTB Mean | MTB SD | MTB Total | Weight | Mean Difference IV, Random, 95% CI |
|---|---|---|---|---|---|---|---|---|
| Silverman et al. 2004 | 0.11 | 0.09 | 19 | 0.14 | 0.11 | 20 | 45.4% | -0.03 [-0.09, 0.03] |
| Yildrim & Kayaalti-Yuksek C 2020 | 0.37 | 0.32 | 10 | 0.61 | 0.28 | 10 | 19.5% | -0.24 [-0.50, 0.02] |
| Yildrim & Kayaalti-Yuksek H 2020 | 0.48 | 0.35 | 10 | 0.5 | 0.22 | 10 | 20.1% | -0.02 [-0.28, 0.24] |
| Yildrim & Kayaalti-Yuksek V 2020 | 0.83 | 0.32 | 10 | 0.54 | 0.41 | 10 | 15.0% | 0.29 [-0.03, 0.61] |
| **Total (95% CI)** | | | **49** | | | **50** | **100.0%** | **-0.02 [-0.17, 0.13]** |

Heterogeneity: $Tau^2 = 0.01$; $Chi^2 = 6.23$, df = 3 (P = 0.10); $I^2 = 52\%$
Test for overall effect: Z = 0.27 (P = 0.78)

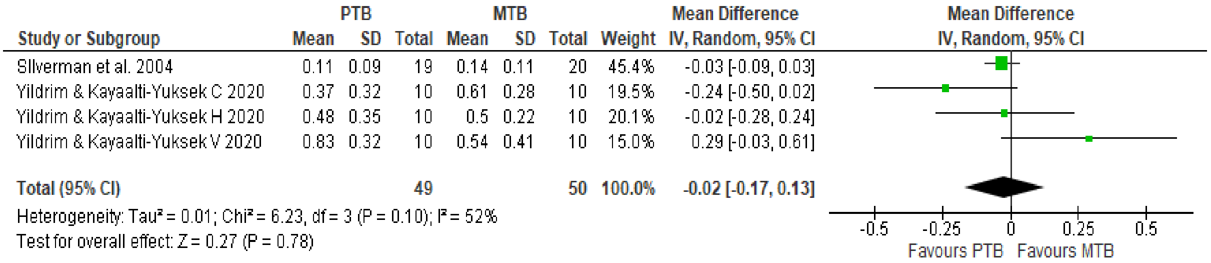


b. End

| Study or Subgroup | PTB Mean | PTB SD | PTB Total | MTB Mean | MTB SD | MTB Total | Weight | Mean Difference IV, Random, 95% CI |
|---|---|---|---|---|---|---|---|---|
| Silverman et al. 2004 | 0.05 | 0.05 | 18 | 0.11 | 0.11 | 20 | 52.1% | -0.06 [-0.11, -0.01] |
| Yildrim & Kayaalti-Yuksek C 2020 | 0.31 | 0.32 | 10 | 0.4 | 0.35 | 10 | 10.2% | -0.09 [-0.38, 0.20] |
| Yildrim & Kayaalti-Yuksek H 2020 | 0.23 | 0.19 | 10 | 0.29 | 0.16 | 10 | 25.7% | -0.06 [-0.21, 0.09] |
| Yildrim & Kayaalti-Yuksek V 2020 | 0.55 | 0.25 | 10 | 0.3 | 0.35 | 10 | 12.0% | 0.25 [-0.02, 0.52] |
| **Total (95% CI)** | | | **48** | | | **50** | **100.0%** | **-0.03 [-0.13, 0.08]** |

Heterogeneity: $Tau^2 = 0.00$; $Chi^2 = 5.09$, df = 3 (P = 0.17); $I^2 = 41\%$
Test for overall effect: Z = 0.50 (P = 0.62)

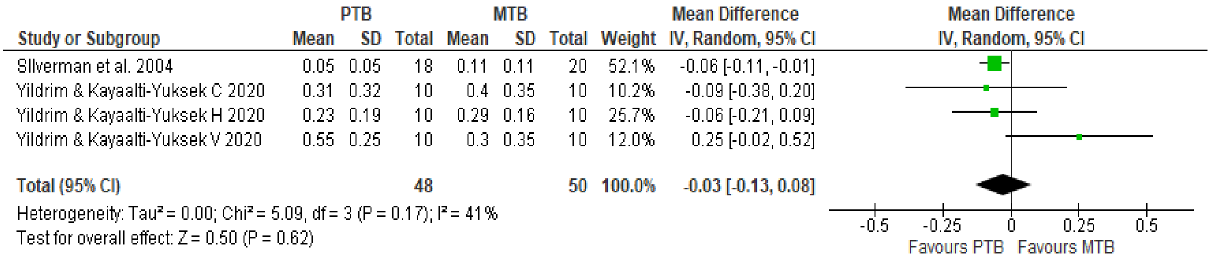


c. Difference

| Study or Subgroup | PTB Mean | PTB SD | PTB Total | MTB Mean | MTB SD | MTB Total | Weight | Mean Difference IV, Fixed, 95% CI |
|---|---|---|---|---|---|---|---|---|
| Yildrim & Kayaalti-Yuksek C 2020 | -0.05 | 0.16 | 10 | -0.21 | 0.19 | 10 | 32.6% | 0.16 [0.01, 0.31] |
| Yildrim & Kayaalti-Yuksek H 2020 | -0.26 | 0.19 | 10 | -0.21 | 0.16 | 10 | 32.6% | -0.05 [-0.20, 0.10] |
| Yildrim & Kayaalti-Yuksek V 2020 | -0.28 | 0.16 | 10 | -0.24 | 0.18 | 10 | 34.7% | -0.04 [-0.19, 0.11] |
| **Total (95% CI)** | | | **30** | | | **30** | **100.0%** | **0.02 [-0.07, 0.11]** |

Heterogeneity: $Chi^2 = 4.59$, df = 2 (P = 0.10); $I^2 = 56\%$
Test for overall effect: Z = 0.49 (P = 0.62)

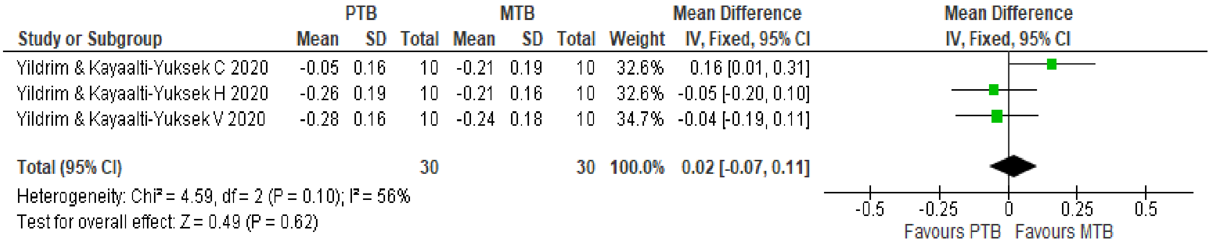

**ONLINE Appendix S9.**

Forest plot using a fixed model of the performed subgroup analysis for the OR mode of action for PTB compared to MTB at <u>studies with a follow-up</u> on plaque index scores on the Q&HPI (49) in children.

a. Baseline

| | PTB | | | MTB | | | | Mean Difference |
|---|---|---|---|---|---|---|---|---|
| **Study or Subgroup** | **Mean** | **SD** | **Total** | **Mean** | **SD** | **Total** | **Weight** | **IV, Fixed, 95% CI** |
| Garcia-Godoy et al. 2011 | 2.64 | 0.69 | 35 | 2.57 | 0.59 | 35 | 0.1% | 0.07 [-0.23, 0.37] |
| Silverman et al. 2004 | 2.17 | 0.42 | 19 | 2.21 | 0.55 | 20 | 0.1% | -0.04 [-0.35, 0.27] |
| Zimmer et al. 1999 | 0 | 0.01 | 12 | 0 | 0.01 | 12 | 99.9% | 0.00 [-0.01, 0.01] |
| **Total (95% CI)** | | | **66** | | | **67** | **100.0%** | **0.00 [-0.01, 0.01]** |

Heterogeneity: Chi² = 0.27, df = 2 (P = 0.87); I² = 0%
Test for overall effect: Z = 0.01 (P = 1.00)

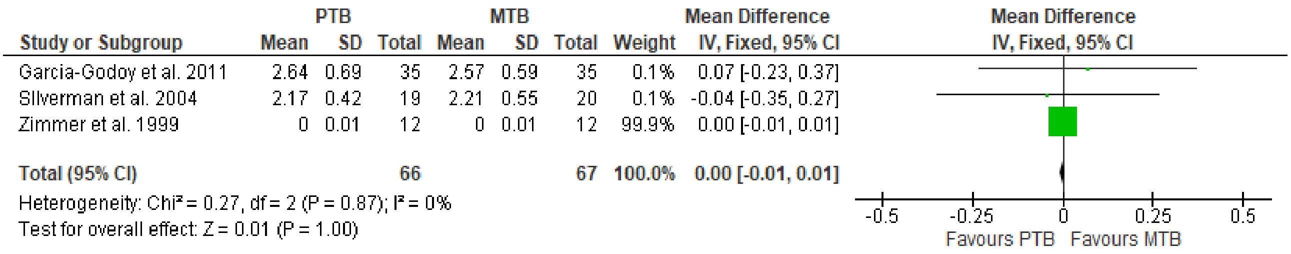


b. End

| | PTB | | | MTB | | | | Mean Difference |
|---|---|---|---|---|---|---|---|---|
| **Study or Subgroup** | **Mean** | **SD** | **Total** | **Mean** | **SD** | **Total** | **Weight** | **IV, Fixed, 95% CI** |
| Garcia-Godoy et al. 2011 | 2.33 | 0.53 | 34 | 2.55 | 0.56 | 32 | 46.5% | -0.22 [-0.48, 0.04] |
| Silverman et al. 2004 | 1.52 | 0.45 | 18 | 1.75 | 0.53 | 20 | 33.2% | -0.23 [-0.54, 0.08] |
| Zimmer et al. 1999 | 2.24 | 0.55 | 12 | 2.28 | 0.44 | 12 | 20.3% | -0.04 [-0.44, 0.36] |
| **Total (95% CI)** | | | **64** | | | **64** | **100.0%** | **-0.19 [-0.37, -0.01]** |

Heterogeneity: Chi² = 0.66, df = 2 (P = 0.72); I² = 0%
Test for overall effect: Z = 2.04 (P = 0.04)

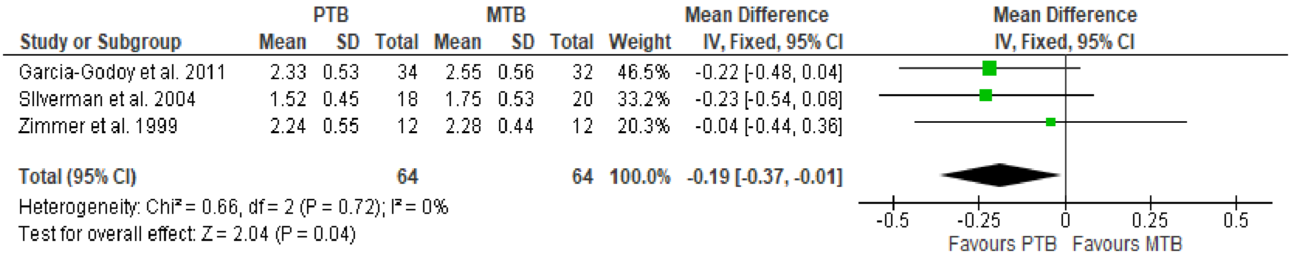


c. Difference

| | PTB | | | MTB | | | | Mean Difference |
|---|---|---|---|---|---|---|---|---|
| **Study or Subgroup** | **Mean** | **SD** | **Total** | **Mean** | **SD** | **Total** | **Weight** | **IV, Fixed, 95% CI** |
| Garcia-Godoy et al. 2011 | -0.31 | 0.58 | 34 | -0.02 | 0.43 | 32 | 72.5% | -0.29 [-0.54, -0.04] |
| Zimmer et al. 1999 | 2.24 | 0.55 | 12 | 2.28 | 0.44 | 12 | 27.5% | -0.04 [-0.44, 0.36] |
| **Total (95% CI)** | | | **46** | | | **44** | **100.0%** | **-0.22 [-0.43, -0.01]** |

Heterogeneity: Chi² = 1.10, df = 1 (P = 0.30); I² = 9%
Test for overall effect: Z = 2.08 (P = 0.04)

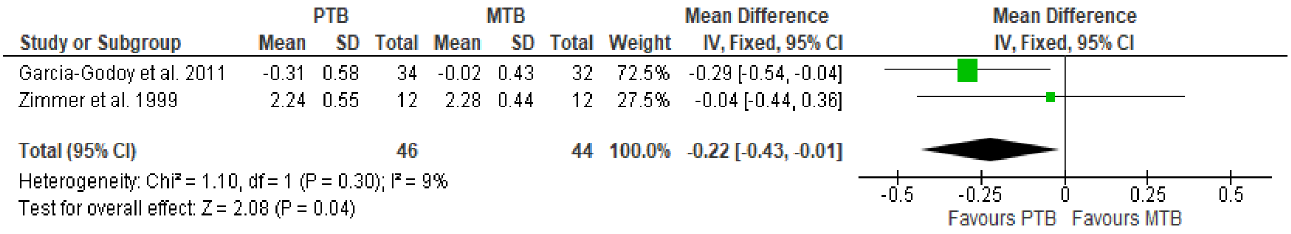

**ONLINE Appendix S10.**

Forest plot using a fixed model of the performed subgroup analysis for PTB compared to MTB at studies with a follow-up and low risk of bias on plaque index scores on the Q&HPI (49) and S&LPI (50) in children.

a. Difference Q&HPI

| | PTB | | | MTB | | | | Mean Difference |
|---|---|---|---|---|---|---|---|---|
| Study or Subgroup | Mean | SD | Total | Mean | SD | Total | Weight | IV, Fixed, 95% CI |
| Garcia-Godoy et al. 2011 | -0.31 | 0.58 | 34 | -0.02 | 0.43 | 32 | 72.5% | -0.29 [-0.54, -0.04] |
| Zimmer et al. 1999 | 2.24 | 0.55 | 12 | 2.28 | 0.44 | 12 | 27.5% | -0.04 [-0.44, 0.36] |
| **Total (95% CI)** | | | **46** | | | **44** | **100.0%** | **-0.22 [-0.43, -0.01]** |

Heterogeneity: Chi² = 1.10, df = 1 (P = 0.30); I² = 9%
Test for overall effect: Z = 2.08 (P = 0.04)

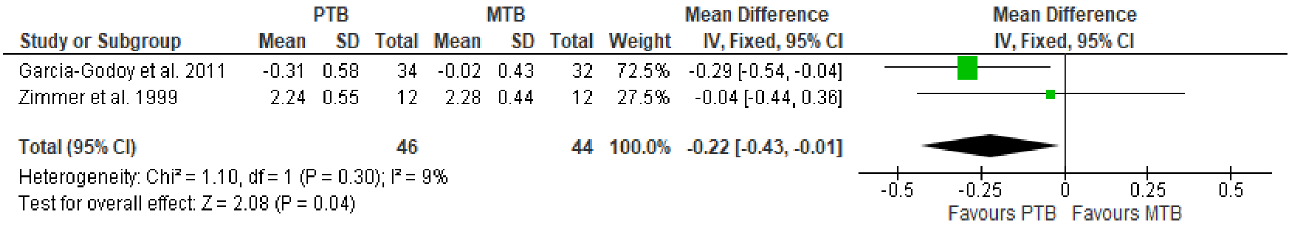


b. Difference S&LPI

| | PTB | | | MTB | | | | Mean Difference |
|---|---|---|---|---|---|---|---|---|
| Study or Subgroup | Mean | SD | Total | Mean | SD | Total | Weight | IV, Fixed, 95% CI |
| Yildrim & Kayaalti-Yuksek C 2020 | -0.23 | 0.25 | 10 | -0.3 | 0.25 | 10 | 36.9% | 0.07 [-0.15, 0.29] |
| Yildrim & Kayaalti-Yuksek H 2020 | -0.44 | 0.25 | 10 | -0.31 | 0.28 | 10 | 32.7% | -0.13 [-0.36, 0.10] |
| Yildrim & Kayaalti-Yuksek V 2020 | -0.71 | 0.28 | 10 | -0.59 | 0.27 | 10 | 30.4% | -0.12 [-0.36, 0.12] |
| **Total (95% CI)** | | | **30** | | | **30** | **100.0%** | **-0.05 [-0.19, 0.08]** |

Heterogeneity: Chi² = 1.93, df = 2 (P = 0.38); I² = 0%
Test for overall effect: Z = 0.78 (P = 0.43)

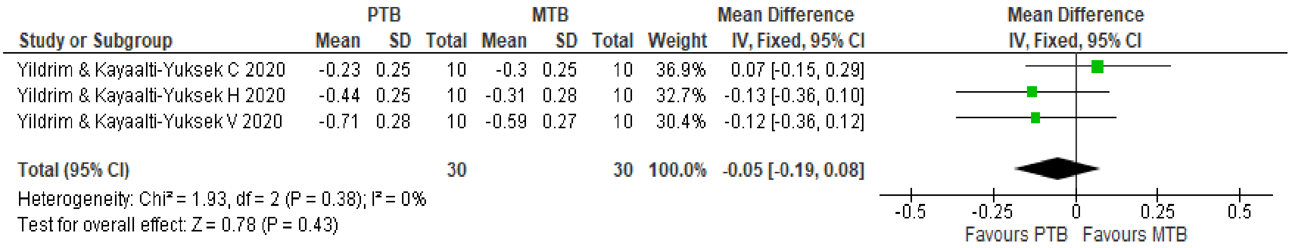

PRISMA 2020 checklist

| Section and Topic | Item # | Checklist item | Location where item is reported |
|---|---|---|---|
| **TITLE** | | | |
| Title | 1 | Identify the report as a systematic review. | 1 |
| **ABSTRACT** | | | |
| Abstract | 2 | See the PRISMA 2020 for Abstracts checklist. | 4 |
| **INTRODUCTION** | | | |
| Rationale | 3 | Describe the rationale for the review in the context of existing knowledge. | 6 |
| Objectives | 4 | Provide an explicit statement of the objective(s) or question(s) the review addresses. | 6 |
| **METHODS** | | | |
| Eligibility criteria | 5 | Specify the inclusion and exclusion criteria for the review and how studies were grouped for the syntheses. | 7-8 |
| Information sources | 6 | Specify all databases, registers, websites, organisations, reference lists and other sources searched or consulted to identify studies. Specify the date when each source was last searched or consulted. | 7 |
| Search strategy | 7 | Present the full search strategies for all databases, registers and websites, including any filters and limits used. | 7 (Table 1) |
| Selection process | 8 | Specify the methods used to decide whether a study met the inclusion criteria of the review, including how many reviewers screened each record and each report retrieved, whether they worked independently, and if applicable, details of automation tools used in the process. | 7 |
| Data collection process | 9 | Specify the methods used to collect data from reports, including how many reviewers collected data from each report, whether they worked independently, any processes for obtaining or confirming data from study investigators, and if applicable, details of automation tools used in the process. | 8 |
| Data items | 10a | List and define all outcomes for which data were sought. Specify whether all results that were compatible with each outcome domain in each study were sought (e.g. for all measures, time points, analyses), and if not, the methods used to decide which results to collect. | 8 |
| | 10b | List and define all other variables for which data were sought (e.g. participant and intervention characteristics, funding sources). Describe any assumptions made about any missing or unclear information. | 8 |
| Study risk of bias assessment | 11 | Specify the methods used to assess risk of bias in the included studies, including details of the tool(s) used, how many reviewers assessed each study and whether they worked independently, and if applicable, details of automation tools used in the process. | 8 |

| Section and Topic | Item # | Checklist item | Location where item is reported |
|---|---|---|---|
| Effect measures | 12 | Specify for each outcome the effect measure(s) (e.g. risk ratio, mean difference) used in the synthesis or presentation of results. | 8 |
| Synthesis methods | 13a | Describe the processes used to decide which studies were eligible for each synthesis (e.g. tabulating the study intervention characteristics and comparing against the planned groups for each synthesis (item #5)). | 7,8 |
| | 13b | Describe any methods required to prepare the data for presentation or synthesis, such as handling of missing summary statistics, or data conversions. | 8 |
| | 13c | Describe any methods used to tabulate or visually display results of individual studies and syntheses. | 9 |
| | 13d | Describe any methods used to synthesize results and provide a rationale for the choice(s). If meta-analysis was performed, describe the model(s), method(s) to identify the presence and extent of statistical heterogeneity, and software package(s) used. | 9 |
| | 13e | Describe any methods used to explore possible causes of heterogeneity among study results (e.g. subgroup analysis, meta-regression). | 9 |
| | 13f | Describe any sensitivity analyses conducted to assess robustness of the synthesized results. | 9 |
| Reporting bias assessment | 14 | Describe any methods used to assess risk of bias due to missing results in a synthesis (arising from reporting biases). | 8,9 |
| Certainty assessment | 15 | Describe any methods used to assess certainty (or confidence) in the body of evidence for an outcome. | 10 |
| **RESULTS** | | | |
| Study selection | 16a | Describe the results of the search and selection process, from the number of records identified in the search to the number of studies included in the review, ideally using a flow diagram. | 11 (Figure 1) |
| | 16b | Cite studies that might appear to meet the inclusion criteria, but which were excluded, and explain why they were excluded. | Appendix S2 |
| Study characteristics | 17 | Cite each included study and present its characteristics. | 11,12 (Table 2) |
| Risk of bias in studies | 18 | Present assessments of risk of bias for each included study. | 12 (Appendix S1) |
| Results of individual studies | 19 | For all outcomes, present, for each study: (a) summary statistics for each group (where appropriate) and (b) an effect estimate and its precision (e.g. confidence/credible interval), ideally using structured tables or plots. | 13 (Appendix S3a&b and S4) |
| Results of syntheses | 20a | For each synthesis, briefly summarise the characteristics and risk of bias among contributing studies. | 11-14 |

| Section and Topic | Item # | Checklist item | Location where item is reported |
|---|---|---|---|
| | 20b | Present results of all statistical syntheses conducted. If meta-analysis was done, present for each the summary estimate and its precision (e.g. confidence/credible interval) and measures of statistical heterogeneity. If comparing groups, describe the direction of the effect. | 13,14 (Table 3a&b and 4a-f) (Appendix S5-10) |
| | 20c | Present results of all investigations of possible causes of heterogeneity among study results. | 11-14 |
| | 20d | Present results of all sensitivity analyses conducted to assess the robustness of the synthesized results. | 13,14 |
| Reporting biases | 21 | Present assessments of risk of bias due to missing results (arising from reporting biases) for each synthesis assessed. | 14 |
| Certainty of evidence | 22 | Present assessments of certainty (or confidence) in the body of evidence for each outcome assessed. | 14 (Table 5) |
| **DISCUSSION** | | | |
| Discussion | 23a | Provide a general interpretation of the results in the context of other evidence. | 15 |
| | 23b | Discuss any limitations of the evidence included in the review. | 15-18 |
| | 23c | Discuss any limitations of the review processes used. | 15 |
| | 23d | Discuss implications of the results for practice, policy, and future research. | 17,18 |
| **OTHER INFORMATION** | | | |
| Registration and protocol | 24a | Provide registration information for the review, including register name and registration number, or state that the review was not registered. | 2,4,7 |
| | 24b | Indicate where the review protocol can be accessed, or state that a protocol was not prepared. | 7 |
| | 24c | Describe and explain any amendments to information provided at registration or in the protocol. | 7 |
| Support | 25 | Describe sources of financial or non-financial support for the review, and the role of the funders or sponsors in the review. | 2 |
| Competing interests | 26 | Declare any competing interests of review authors. | 2 |
| Availability of data, code and other materials | 27 | Report which of the following are publicly available and where they can be found: template data collection forms; data extracted from included studies; data used for all analyses; analytic code; any other materials used in the review. | Online Appendix |

PRISMA 2020 for abstracts checklist

| **Section and Topic** | **Item #** | **Checklist item** | **Reported (Yes/No)** |
|---|---|---|---|
| **TITLE** | | | |
| Title | 1 | Identify the report as a systematic review. | Yes |
| **BACKGROUND** | | | |
| Objectives | 2 | Provide an explicit statement of the main objective(s) or question(s) the review addresses. | Yes |
| **METHODS** | | | |
| Eligibility criteria | 3 | Specify the inclusion and exclusion criteria for the review. | Yes |
| Information sources | 4 | Specify the information sources (e.g. databases, registers) used to identify studies and the date when each was last searched. | Yes |
| Risk of bias | 5 | Specify the methods used to assess risk of bias in the included studies. | Yes |
| Synthesis of results | 6 | Specify the methods used to present and synthesise results. | Yes |
| **RESULTS** | | | |
| Included studies | 7 | Give the total number of included studies and participants and summarise relevant characteristics of studies. | Yes (comparisons instead of participants) |
| Synthesis of results | 8 | Present results for main outcomes, preferably indicating the number of included studies and participants for each. If meta-analysis was done, report the summary estimate and confidence/credible interval. If comparing groups, indicate the direction of the effect (i.e. which group is favoured). | Yes |
| **DISCUSSION** | | | |
| Limitations of evidence | 9 | Provide a brief summary of the limitations of the evidence included in the review (e.g. study risk of bias, inconsistency and imprecision). | Yes |
| Interpretation | 10 | Provide a general interpretation of the results and important implications. | Yes |
| **OTHER** | | | |
| Funding | 11 | Specify the primary source of funding for the review. | Yes |
| Registration | 12 | Provide the register name and registration number. | Yes |
